# Machine Learning-Driven Prediction of Formability and Cubic Phase in Compositionally Complex ABO_3_ Perovskite Oxides

**DOI:** 10.3390/ma19184015

**Published:** 2026-09-21

**Authors:** Jie Zhao, Xiaoyan Wang

**Affiliations:** 1 College of Chemical Engineering, Nanjing Tech University, Nanjing 211816, China; 2 School of Computer Science, Nanjing Audit University, Nanjing 211815, China

**Keywords:** perovskite oxides, machine learning, formability, cubic structure, high-throughput screening

## Abstract

Perovskite oxides (ABO_3_) are widely investigated for energy-related applications due to their compositional flexibility and tunable physicochemical properties. However, identifying formable and cubic perovskite oxides from the vast compositional space remains challenging using conventional approaches, especially for doped perovskite oxides. In this work, a two-stage machine learning framework is developed to accelerate the discovery of formable and cubic perovskite oxides in doped ABO_3_ compositions. A dataset comprising 1502 ABO_3_ compounds was compiled from published literature. Compositional features derived from elemental properties were used to describe them, and feature selection was employed to identify the most relevant descriptors. It was found that perovskite formability and cubic structure were greatly determined by radius-based and volume-related descriptors, respectively. Compared with other algorithms, three ensemble learning algorithms, including Random Forest (RF), Extreme Gradient Boosting (XGB), and Light Gradient Boosting Machine (LGBM), displayed better performance. For formability prediction, XGB yielded the best results with an accuracy of 0.910 and an F1 score of 0.937, while for cubic structure prediction, it achieved an accuracy of 0.924 and an F1 score of 0.889. The trained models were subsequently applied to screen 710,269 virtual ABO_3_ compositions, identifying 505,698 and 217,596 candidates predicted to be formable and cubic, respectively, with 189,247 satisfying both criteria. This study provides an efficient framework for high-throughput screening and rational design of advanced perovskite oxides.

## 1. Introduction

**Figure 1:**
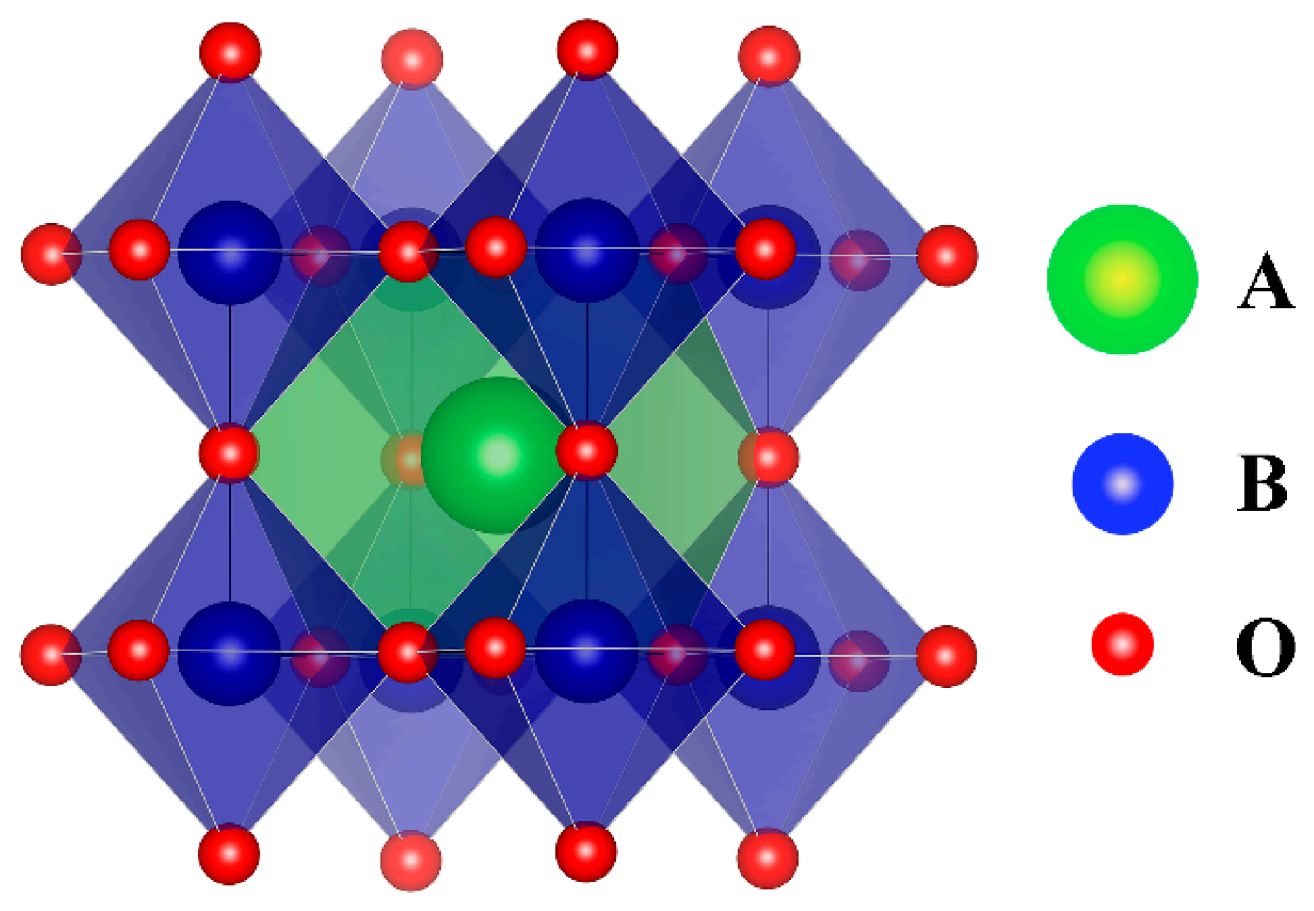
Crystal structure of cubic perovskite oxides [8].

In recent decades, perovskite oxides (ABO_3_) have attracted extensive attention as a structurally versatile class of functional materials [1,2,3]. In the ideal cubic perovskite structure (Figure 1), the larger A-site cation occupies a 12-fold coordinated site, while the smaller B-site cation resides within an oxygen octahedron [4,5]. Owing to the geometric flexibility of the corner-sharing BO_6_ framework, small variations in ionic radii, oxidation states, or electronic configurations can induce octahedral tilting, cation displacement, and symmetry lowering to tetragonal, orthorhombic, or rhombohedral phases [6,7]. Therefore, perovskite oxides exhibit remarkable structural diversity across a vast compositional space.

This structural flexibility makes it challenging to determine whether a given composition can form a perovskite phase and, if so, which crystal symmetry it will adopt. The ABO_3_ framework tolerates a wide range of cation combinations, and subtle compositional variations may shift formability among competing polymorphs or even favor non-perovskite structures [9,10]. Traditionally, perovskite formability has been evaluated using geometric criteria derived from ionic radii. The most widely used descriptor is the Goldschmidt tolerance factor (*t*),

(1) $$t=\frac{\left(r_{A}+r_{O}\right)}{\sqrt{2}(r_{B}+r_{O})},$$

where *r_A_*, *r_B_*, and *r_O_* denote the ionic radii of the A-site cation, B-site cation, and oxygen anion, respectively [11]. Another geometric parameter, the octahedral factor,

(2) $$\mu \;=\frac{r_{B}}{r_{O}},$$

has been proposed as a criterion for cubic perovskite formability [12]. Cubic perovskite structures are typically found when the octahedral factor falls within the range 0.414 < *μ* < 0.732. Also, a modified tolerance factor τ was recently introduced to improve the prediction of perovskite formability:

(3) $$\tau \;=\;\frac{r_{O}}{r_{B}}\;-\;n_{A}(n_{A}-\frac{r_{A}/r_{B}}{\ln(r_{A}/r_{B})}),$$

where *n_A_* denotes the oxidation state of the A-site cation. ABO_3_ compounds satisfying *τ* < 4.18 are likely to form perovskite oxides [13]. In addition to these descriptors, a combined factor (*μ* + *t*)^η^ was proposed to predict cubic perovskites [14]. Despite their usefulness, these descriptors remain simplified geometric heuristics and may not fully capture the complex chemical interactions governing perovskite formation and structural symmetry, especially in multicomponent systems. For instance, although perovskite oxides are generally expected to form when *t* lies within the range of 0.75–1.05 [15], this criterion is not universally reliable. Some ABO_3_ compounds with intermediate *t* values fail to adopt the perovskite structure, highlighting the limitations of such descriptors [15,16]. Beyond formability, accurately identifying the crystal symmetry is equally important, as it strongly influences material properties. In particular, cubic perovskite oxides often exhibit superior electronic conductivity, oxygen permeability, and capacitance compared with their lower-symmetry counterparts [17,18,19,20]. Therefore, reliable screening of both formable and cubic perovskite oxides is critical.

Moreover, the enormous compositional space generated by A-site and B-site substitutions further renders exhaustive experimental investigation impractical. For instance, even when restricting to the stoichiometrically simple equimolar double-substituted case (A_0.5_A’_0.5_B_0.5_B’_0.5_O_3_), more than 28 million distinct perovskite oxide compositions are theoretically possible [21]. This combinatorial explosion severely limits purely experimental approaches. Consequently, first-principles calculations based on density functional theory have been employed to screen formable perovskite oxides and predict perovskite crystal structures, yet their computational cost limits their applicability in large-scale compositional screening [22,23]. These challenges highlight the need for efficient and scalable approaches capable of identifying synthesizable and cubic perovskite oxides across high-dimensional chemical spaces.

Recently, machine learning (ML) has been widely employed to predict the formability and crystal structure of perovskite oxides. For example, Zhao et al. [4] combined ML with constraint satisfaction rules to screen formable and stable ABO_3_ compounds, revealing that formability is primarily governed by structural features, whereas stability is strongly influenced by B-site elemental characteristics. 1373 formable perovskite oxides were screened from 2229 ABO_3_ combinations. Wang et al. [5] further introduced multi-label classification to simultaneously predict perovskite formability and stability, overcoming the limitations of conventional single-label approaches. An optimized model achieved a subset accuracy of 0.932, and 42 formable and stable perovskite oxides were identified from 2226 virtual perovskite combinations. Chen et al. [24] developed an ML framework for predicting perovskite formability based on compositional and structural features. Compressed sensing was employed to construct two interpretable descriptors, which, together with the *t*, *μ*, B-site Mendeleev number, and B-site atomic volume, were used in a gradient-boosted decision tree model. 591 previously unexplored ABO_3_ compounds were identified as formable perovskite oxides. Zhai et al. [15] proposed a function-confined ML approach to discover new fractionally doped perovskite oxides (FDPOs) for solar thermochemical hydrogen production, achieving a prediction accuracy of 95.4% using only 632 experimental data points. The model was further validated against 36 independent experimental data points and enabled the identification and synthesis of 11 new FDPO compositions, seven of which showed potential for solar thermochemical hydrogen production. Jarin et al. [25] developed multiple ML models to classify perovskite crystal structures and predict lattice parameters using basic atomic characteristics. Among the evaluated models, the genetic algorithm (GA)-assisted neural network achieved an average classification accuracy of approximately 88%, while the GA-assisted support vector regression model reached an average accuracy of about 95% for lattice-parameter prediction, demonstrating the potential of ML to accelerate perovskite materials discovery. High-throughput ML frameworks have also been developed to explore large compositional spaces, enabling the identification of hundreds of promising candidates for experimental validation [26,27].

Despite these advances, most existing studies focus on undoped single perovskite compounds based on idealized ABO_3_ datasets, limiting their applicability to real-world systems where doping and compositional complexity are ubiquitous. Furthermore, although several studies have addressed crystal structure prediction, efforts specifically focused on identifying cubic perovskite oxides in undoped perovskites and compositionally complex systems remain relatively scarce.

Hence, in this study, an ML framework is developed to screen formable and cubic perovskite oxides across a broad compositional space. By constructing an input dataset of experimentally reported ABO_3_ compounds, we formulate the problem as a two-stage supervised learning task that integrates formability classification with subsequent cubic prediction. Compositional descriptors are employed to represent each compound in the dataset, capturing both geometric and physicochemical characteristics of the A- and B-site elements. After feature selection, multiple ML algorithms are evaluated and compared, and the optimal algorithms are selected to train the final models, which are then applied to screen unseen compositions generated by a constraint satisfaction technique proposed in our previous study [4]. The novelty of the present work lies in integrating perovskite formability prediction and cubic-structure classification into a unified sequential screening framework for compositionally complex perovskites, identifying distinct descriptor–structure relationships associated with these two structural criteria, and explicitly considering oxygen non-stoichiometry in the generation of virtual ABO_3−δ_ compositions. The resulting framework enables high-throughput screening of a large compositional space and provides a structurally prioritized candidate pool for further investigation.

## 2. Methods

### 2.1. Dataset Construction

**Figure 2:**
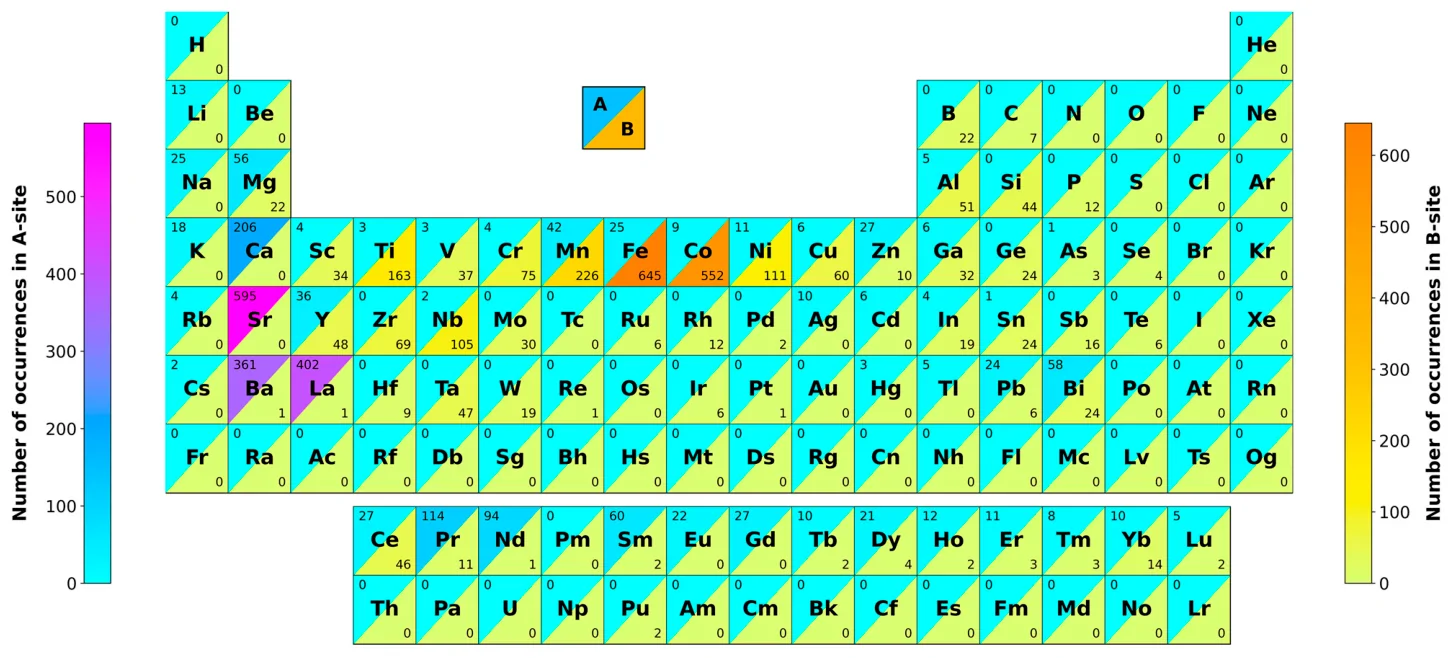
The distribution of the A-site and B-site elements of the doped and undoped ABO_3_ compounds in the dataset.

A dataset comprising 1502 ABO_3_-type compounds (Table S1) was collected from published literature. In these compounds, A-site and/or B-site doped and undoped ABO_3_-type compounds were included. The maximum number of cations on the A-site and B-site is 6 and 4, respectively. Each compound was labelled with two targets: formability (1 for perovskite and 0 for non-perovskite) and crystal type (1 for cubic and 0 for non-cubic). In the dataset, there were 1078 perovskite oxides and 424 non-perovskite compounds, or 508 cubic perovskite oxides and 994 non-cubic compounds. It has to be noted that ABO_3_ compounds with mixed perovskite phases were regarded as non-perovskites and non-cubic phases in this study. For example, La_0.1_Ba_0.9_Co_1−y_Fe_y_O_3−δ_ (y = 0.1 and 0.2) were not labelled as perovskite oxides, since there were secondary phases in addition to the main phase in these compounds [28]. Furthermore, every compound was described with 456 elemental features. The features can be categorized into three groups: A-site elemental features, B-site elemental features, and composition-based features, with each group comprising 152 features. Within each group, the features are derived from 38 elemental attributes (Table S2) by calculating four statistical descriptors—namely, the compositional average, difference, maximum value, and minimum value. The difference was calculated as the range between the maximum and minimum values. Figure 2 illustrates the distribution of elements occupying the A- and B-sites within the dataset. The top-left triangle represents A-site elements, and the bottom-right triangle represents B-site elements, with both color gradients and numerical annotations indicating their occurrence frequencies.

### 2.2. Feature Reduction and Descriptor Selection

To identify the most informative features, a feature selection procedure was implemented. To prevent data leakage and ensure robustness, the dataset was first partitioned into training (80%) and testing (20%) sets using stratified sampling to preserve class distributions. An ML pipeline was then constructed, incorporating the Synthetic Minority Over-sampling Technique (SMOTE) to address class imbalance [29], followed by feature selection using SelectFromModel with a Random Forest (RF) classifier from the scikit-learn package [15]. An RF model was then trained on the selected features to further investigate feature importance and feature-response relationships using SHapley Additive exPlanations (SHAP) [30]. Model performance was evaluated using 5-fold cross-validation (CV) on the training set, with the F1 score (Equation (7)) and ROC-AUC (receiver operating characteristic-area under the curve) as evaluation metrics rather than accuracy (Equation (4)). The F1 score, defined as the harmonic mean of precision (Equation (5)) and recall (Equation (6)), provides a balanced assessment of classification performance, particularly for imbalanced datasets [31]. The ROC-AUC evaluates the discriminative capability of the model by measuring its ability to distinguish between classes across varying decision thresholds [32]. The pipeline was subsequently trained on the entire training set, and its generalization performance was assessed on the held-out test set. The selected features were then extracted for use in subsequent modeling. In the following equations, TP, TN, FP, and FN denote true positive, true negative, false positive, and false negative, respectively.

(4) $$\mathrm{Accuracy}=\frac{\mathrm{TP}+\mathrm{TN}}{\mathrm{TP}+\mathrm{TN}+\mathrm{FP}+\mathrm{FN}}$$

(5) $$\mathrm{Precision}=\frac{\mathrm{TP}}{\mathrm{TP}+\mathrm{FP}}\;$$

(6) $$\mathrm{Recall}=\frac{\mathrm{TP}}{\mathrm{TP}+\mathrm{FN}}\;$$

(7) $$F1=2\;\times \;\frac{\mathrm{Precision}\;\times \;\mathrm{Recall}}{\mathrm{Precision}+\mathrm{Recall}}$$

### 2.3. Model Development and Hyperparameter Optimization

Seven algorithms were evaluated on the selected features: Logistic Regression (LR), RF, Support Vector Machine (SVM), K-Nearest Neighbors (KNN), Gradient Boosting (GB), XGBoost (XGB), and LightGBM (LGBM) [33,34]. During algorithm selection, the default hyperparameters of these algorithms were used, and performance was assessed using 5-fold CV with F1 score and ROC-AUC as metrics. Based on the mean F1 score, RF, XGB, and LGBM were identified as the best-performing models and were therefore selected for further study.

Hyperparameter optimization was conducted for the top three models using GridSearchCV with 5-fold CV, optimizing the F1 score. The tuned hyperparameters are summarized in Tables S3 and S4. The optimized models were evaluated on the test set, producing confusion matrices, ROC curves, precision-recall (PR) curves, and prediction probability distributions, along with performance metrics including accuracy, precision, recall, F1 score, ROC-AUC, and PR-AUC. CV results were reported to ensure robustness, and the best-performing models were retained for generalization.

### 2.4. Model Generalization

To screen formable and cubic perovskite oxides from unexplored compositions, doped ABO_3-δ_ combinations were generated by a constraint satisfaction technique [4] from a pool of 7 A-site cations (Ba^2+^, Ca^2+^, Gd^3+^, La^3+^, Pr^3+^, Pr^4+^, Sr^2+^) and 14 B-site cations (Fe^3+^, Fe^4+^, Cr^3+^, Cr^4+^, Co^3+^, Co^4+^, Mn^3+^, Mn^4+^, Mo^5+^, Mo^6+^, Nb^5+^, Ta^5+^, Ti^3+^, Ti^4+^). Cations with different oxidation states of the same element were regarded as distinct species during virtual composition generation to accurately represent charge diversity in doped ABO_3_ systems. Given the combinatorial complexity of the system, the number of components occupying the A-site was restricted to two, while up to four components were allowed for the B-site. The fractional occupancies were discretized with a step size of 0.1. Any overlap of elements between the A-site and B-site was prohibited.

Unlike ideal stoichiometric perovskites, global charge neutrality was not strictly enforced. Instead, oxygen non-stoichiometry (δ) was introduced to compensate for charge imbalance, defined as:

(8) $$\delta \;=\;\frac{6\;-\;(Z_{A}\;+\;Z_{B})}{2},$$

where Z_A_ and Z_B_ represent the average oxidation states of the A-site and B-site cations, respectively. The average oxidation state was calculated as the composition-weighted mean of the constituent elements. Compositions satisfying 0 ≤ δ ≤ 0.5 were retained, as this range reflects realistic oxygen vacancy concentrations commonly observed in defective perovskite oxides [35,36]. In addition, to eliminate geometrically unfavorable compositions, *t* was employed as a preliminary screening criterion during combination generation. Only candidates within the range of 0.75 ≤ *t* ≤ 1.10 were retained, as this interval is usually associated with the formation of perovskite structures [35].

Finally, the hyperparameter-optimized models based on RF, XGB, and LGBM were applied to screen formable and cubic perovskite oxides from the doped ABO_3-δ_ compositional space. Compositions predicted as formable by all three models were identified as formable perovskite oxides, while those consistently classified as cubic by the three models were regarded as cubic perovskite oxides.

## 3. Results and Discussion

### 3.1. Feature Insights for Formability and Cubic Prediction

**Figure 3:**
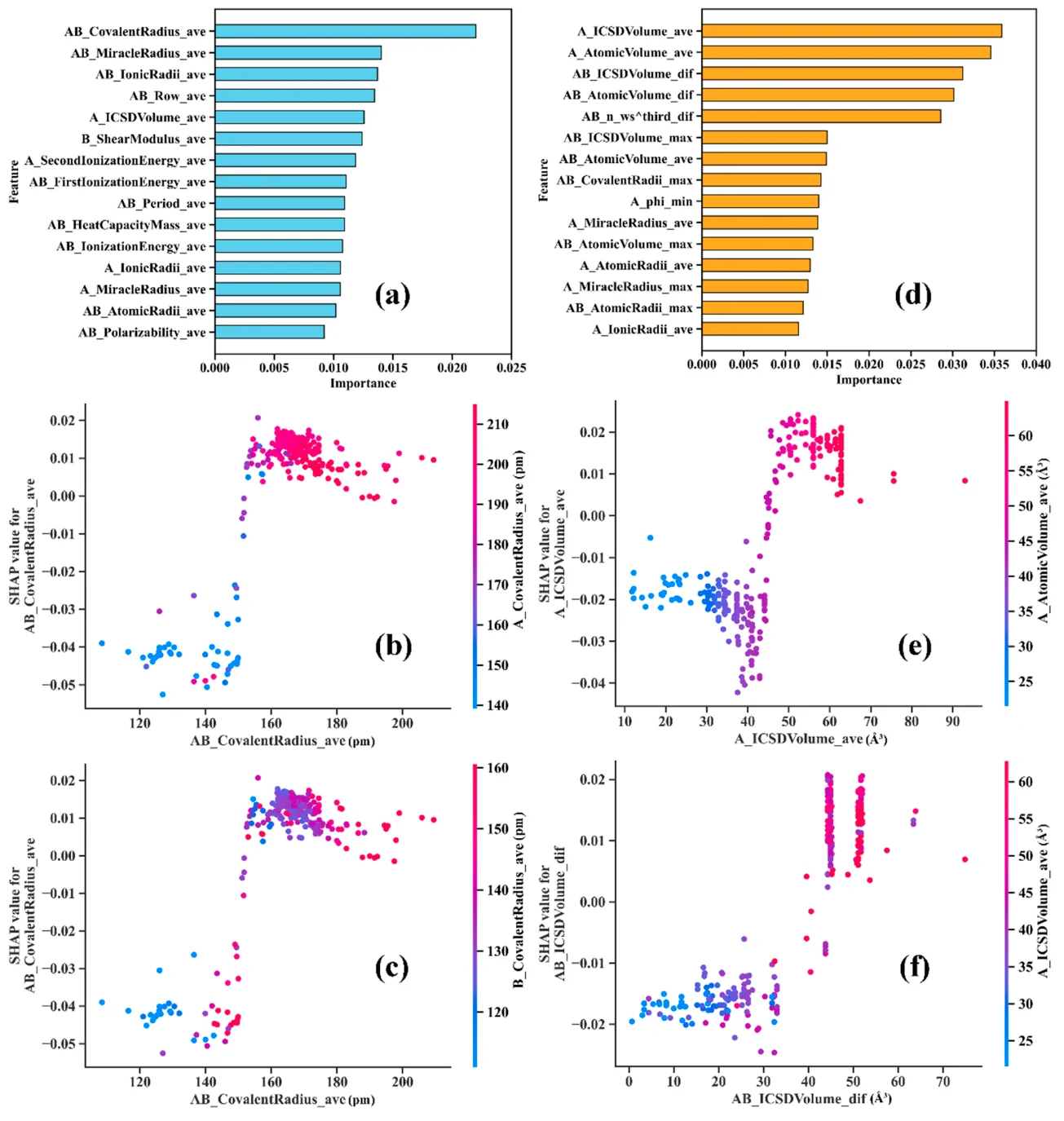
Top 15 feature importance and SHAP interpretation results for formability (**a**–**c**) and cubic phase prediction (**d**–**f**).

Figure 3 presents the feature importance and SHAP interpretation derived from RF models trained on the selected descriptors after feature selection. Shown in Figure 3a is the importance ranking of the top 15 features for perovskite formability prediction. Among these, AB_CovalentRadius_ave is identified as the most influential descriptor, followed by AB_MiracleRadius_ave and AB_IonicRadii_ave, emphasizing the significant role of geometric factors in governing perovskite formability and consistent with classical structure-formability relationships, such as *t*. Particularly, 10 of the 15 features are averaged descriptors that involve both A-site and B-site elements, suggesting that the interplay between the two sites plays a critical role in determining structural formability.

In contrast to the importance of radius-based descriptors in formability prediction, the most important features for cubic perovskite prediction are volume-related (Figure 3d), including A_ICSDVolume_ave, A_AtomicVolume_ave, AB_ICSDVolume_dif, and AB_AtomicVolume_dif. This shift indicates that the stabilization of the cubic phase is more sensitive to the lattice volume rather than local geometric compatibility. In the ideal cubic structure, the A-site cation resides in a 12-fold coordinated cuboctahedral cavity formed by corner-sharing BO_6_ octahedra. A sufficiently large A-site volume is therefore required to stabilize this highly symmetric framework and suppress octahedral tilting distortions. A balanced volume relationship between A- and B-site cations (AB_ICSDVolume_dif and AB_AtomicVolume_dif) is also essential for cubic phase retention.

Figure 3b,c display SHAP dependence plots of the most important descriptor AB_CovalentRadius_ave for perovskite formability prediction. The *x*-axis represents the feature values, while the *y*-axis denotes the corresponding SHAP values, and the color scale reflects the values of the interacting features, namely A_CovalentRadius_ave and B_CovalentRadius_ave, respectively. It can be seen from Figure 3b that compositions with lower AB_CovalentRadius_ave values (≈110–150 pm) tend to exhibit negative SHAP values, indicating a reduced likelihood of forming the perovskite phase, whereas higher values correspond to positive contributions, favoring perovskite formation. Furthermore, when AB_CovalentRadius_ave contributes positively to the prediction, A_CovalentRadius_ave typically assumes relatively large values (>180 pm), while B_CovalentRadius_ave spans a broader range (Figure 3c). This implies that a larger A-site cation size is particularly favorable for perovskite formation, whereas the B-site cation size exhibits greater flexibility, consistent with the geometric requirements of the perovskite framework.

The SHAP dependence plot in Figure 3e illustrates the effect of A_ICSDVolume_ave on the prediction of cubic perovskite oxides. At low values of A_ICSDVolume_ave (≈10–40 Å^3^), the SHAP values are all negative, indicating that insufficient A-site volume strongly disfavors the formation of the cubic phase. As the A-site volume increases to an intermediate range (≈35–50 Å^3^), the SHAP values reach a minimum and then rapidly increase, suggesting a transition region where the structural preference shifts. For larger values (>50–60 Å^3^), the SHAP values become positive, which means that sufficiently large A-site volumes stabilize the cubic structure. The color gradient reveals a strong correlation between A_ICSDVolume_ave and A_AtomicVolume_ave. Higher A_AtomicVolume_ave values are associated with positive SHAP values, reinforcing the conclusion that larger A-site cations favor cubic symmetry. However, once a critical A-site volume threshold is reached, further increases provide diminishing stabilization effects.

Figure 3f illustrates the influence of AB_ICSDVolume_dif on the prediction of cubic perovskite oxides. When AB_ICSDVolume_dif lies in the range of 0–35 Å^3^, the SHAP values are negative, indicating that small volume differences between A-site and B-site elements are unfavorable for cubic phase stabilization. As it increases to 42–45 Å^3^, the SHAP values rapidly transition from negative to positive, suggesting a critical region where the likelihood of forming a cubic structure significantly increases. Beyond this threshold, the SHAP values become consistently positive, indicating that sufficiently large volume differences promote cubic symmetry. The color distribution further reveals that larger A_ICSDVolume_ave values are predominantly associated with positive SHAP values, especially in the high AB_ICSDVolume_dif region.

### 3.2. Comparative Performance of ML Models

**Figure 4:**
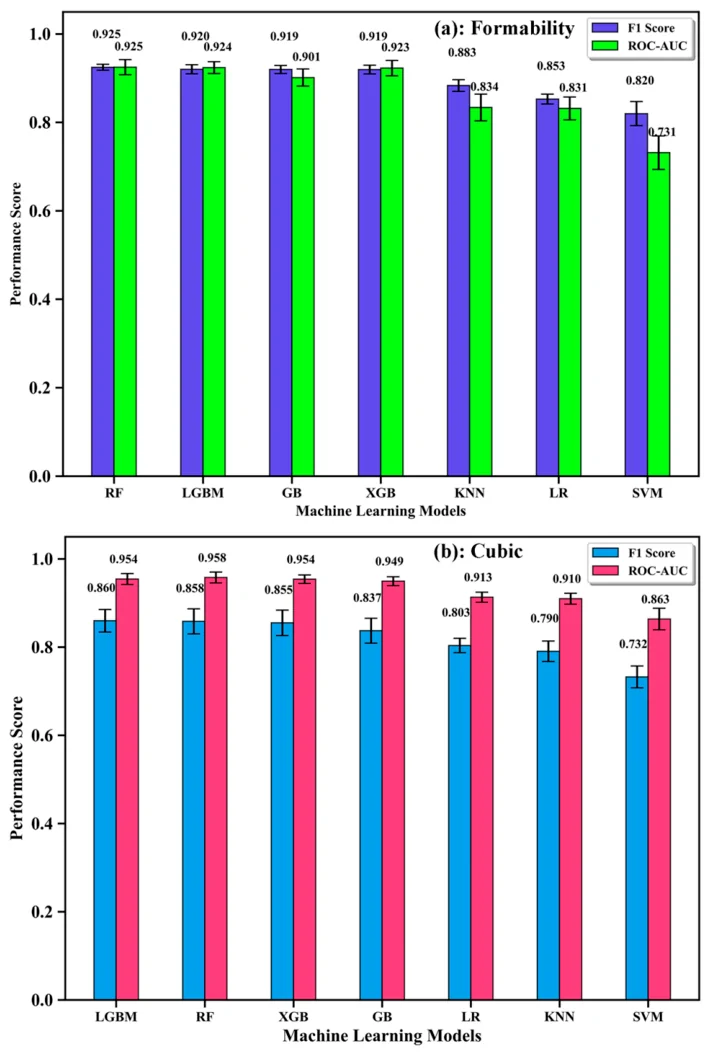
Performance comparison of ML models for perovskite oxide formability (**a**) and cubic phase (**b**) classification.

Figure 4 compares the performance of seven algorithms on the prediction of formable and cubic perovskite oxides. For formability prediction (Figure 4a), tree-based ensemble methods (RF, LGBM, and XGB) achieve superior performance, with F1 scores and ROC-AUC values reaching 0.925. These models outperformed simpler algorithms, such as KNN, LR, and SVM, which show lower performance (F1 score ≈ 0.820–0.883). The high scores of ensemble models suggest their ability to capture complex, non-linear interactions among compositional features, which is crucial for accurately predicting perovskite formability. Additionally, the error bars for the three top models indicate their stable performance across CV folds, demonstrating their robustness and generalizability.

In the case of cubic structure prediction (Figure 4b), LGBM, RF, and XGB again lead, achieving ROC-AUC values around 0.954–0.958 and F1 scores of approximately 0.855–0.860. The performance of KNN and LR is lower, with F1 scores around 0.800 and ROC-AUC values of approximately 0.910. SVM performs the worst, with an F1 score of 0.732. These results confirm that ensemble methods excel in predicting the cubic structure of perovskites. Therefore, LGBM, RF, and XGB algorithms were finally selected to train the final models for formable and cubic perovskite prediction.

**Figure 5:**
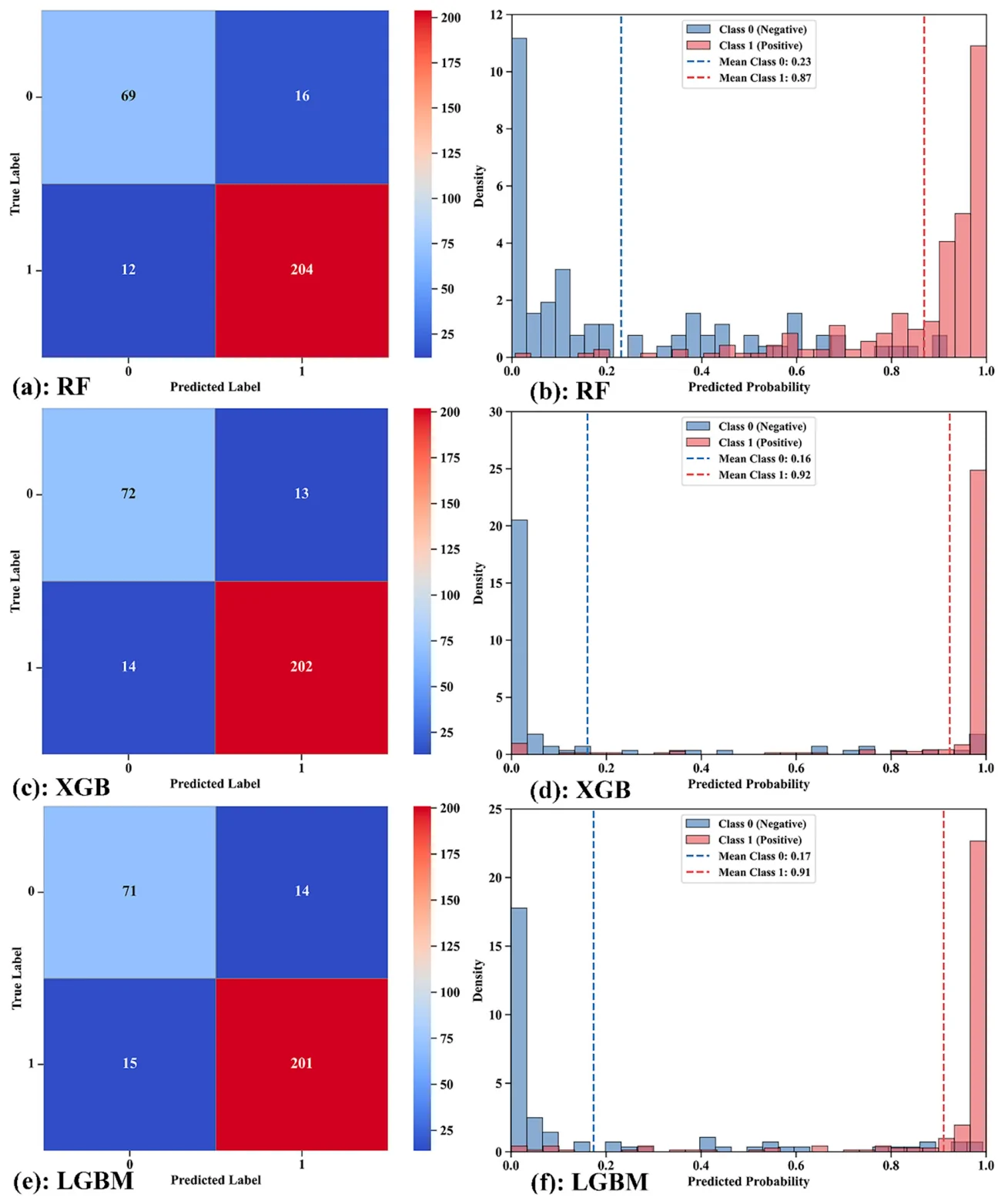
Confusion matrices (**a**,**c**,**e**) and predicted probability distributions (**b**,**d**,**f**) of the final RF, XGB, and LGBM models for perovskite formability prediction, respectively.

Figure 5 compares the performance of the final RF, XGB, and LGBM models for predicting perovskite oxide formability, using confusion matrices (Figure 5a,c,e) and predicted probability distributions (Figure 5b,d,f), respectively. Quantitative performance metrics of these models are summarized in Table 1. As we can see, all three models exhibit strong classification capability, achieving high accuracy in identifying both formable and non-formable compounds. More specifically, the RF model correctly classifies 69 negative and 204 positive samples. It achieves an accuracy of 0.907, with a precision, recall, and F1 score of 0.927, 0.944, and 0.936, respectively. XGB shows a slight improvement over RF, correctly identifying 72 non-perovskites (Class 0) and 202 perovskites (Class 1), with reduced misclassification rates. Its accuracy, precision, recall, and F1 score reach 0.910, 0.940, 0.935, and 0.937, respectively. LGBM demonstrates comparable performance to both RF and XGB, with similar numbers of correct and incorrect predictions, highlighting the consistency of ensemble-based approaches. The corresponding accuracy, precision, recall, and F1 score are 0.904, 0.935, 0.931, and 0.933, respectively.

The predicted probability distributions further confirm the reliability of these models. For all three algorithms, Class 0 samples are predominantly assigned low probabilities, whereas Class 1 samples are associated with high probabilities, demonstrating clear separation between the two classes. To be specific, XGB achieves the most distinct separation, with mean predicted probabilities of 0.16 for Class 0 and 0.92 for Class 1, compared to 0.23/0.87 for RF and 0.17/0.91 for LGBM. This indicates that XGB provides more confident and discriminative predictions.

The ROC and PR curves (Figure S1) for the three models also demonstrate their excellent discriminative capability and robust classification performance in distinguishing formable from non-formable ABO_3_ compositions. The ROC curves (Figure S1a,c,e) of all models are strongly concentrated toward the upper-left corner, indicating high true-positive rates achieved at low false-positive rates. The ROC-AUC values are all above 0.960. PR analysis emphasizes the balance between precision and recall, especially informative for datasets that may exhibit class imbalance [37]. The PR curves (Figure S1b,d,f) of all three models maintain high precision over a broad recall range, with PR-AUC values exceeding 0.980. XGB achieves the best overall PR performance of 0.985.

**Figure 6:**
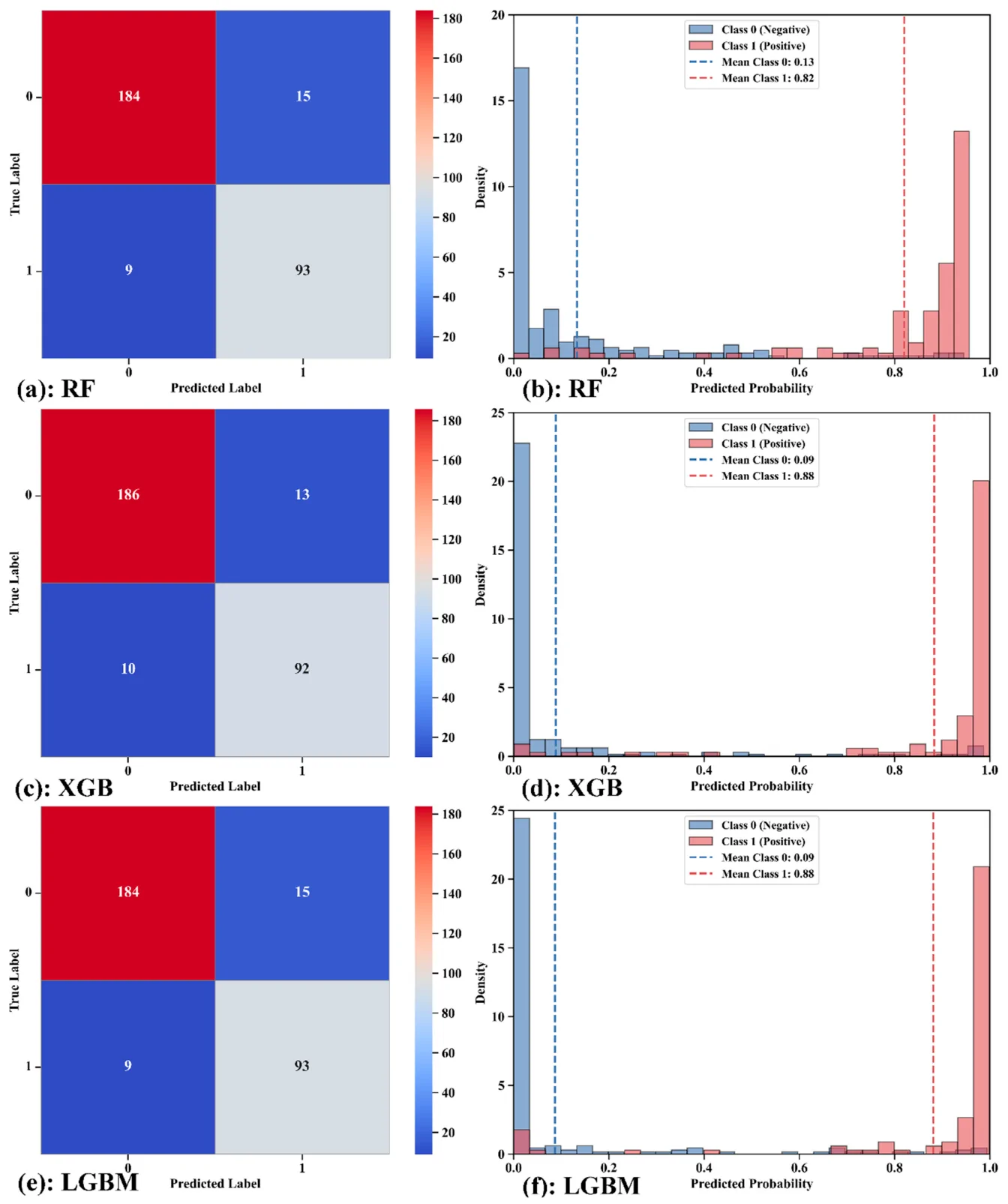
Confusion matrices (**a**,**c**,**e**) and predicted probability distributions (**b**,**d**,**f**) of the final RF, XGB, and LGBM models for cubic perovskite prediction, respectively.

Figure 6 presents the performance of the final RF, XGB, and LGBM models for cubic perovskite prediction, evaluated using confusion matrices and predicted probability distributions. The corresponding accuracy, precision, recall, and F1 scores are summarized in Table 2. It can be seen that all three models demonstrate strong and consistent classification performance. For example, the RF model (Figure 6a) correctly classifies 184 non-cubic and 93 cubic samples. It attains an accuracy of 0.920, with a precision, recall, and F1 score of 0.861, 0.912, and 0.886, respectively. The XGB model (Figure 6c) exhibits a slight performance improvement, correctly identifying 186 non-cubic and 92 cubic samples, suggesting enhanced robustness and generalization ability. Its accuracy, precision, recall, and F1 score are 0.924, 0.876, 0.902, and 0.889, respectively. In terms of the LGBM model (Figure 6e), it shows performance comparable to XGB, correctly classifying 184 non-cubic and 93 cubic samples. The corresponding accuracy, precision, recall, and F1 score are 0.920, 0.861, 0.912, and 0.886, respectively. These results suggest that cubic phase prediction, while more challenging than formability classification, can still be effectively captured by ensemble-based models with high reliability.

The predicted probability distributions provide additional insight into model confidence. For all models, non-cubic compounds are predominantly assigned low probabilities, whereas cubic perovskite oxides are associated with high probabilities. Specifically, XGB (Figure 6d) and LGBM (Figure 6f) exhibit sharper probability separation than RF (Figure 6b), with mean predicted probabilities of 0.09 for the non-cubic class and 0.88 for cubic perovskites, compared to 0.13 and 0.82 for RF. This suggests that boosting-based models not only improve classification accuracy but also yield more confident and discriminative predictions. These results demonstrate that ensemble learning methods are highly effective for predicting cubic perovskite oxides, especially for XGB and LGBM algorithms.

The ROC and PR curves of the three models for cubic structure prediction were also recorded, as shown in Figure S2. Among them, XGB exhibits the best performance, closely followed by LGBM, whereas RF remains highly competitive. The ROC-AUC values are 0.957 for RF (Figure S2a), 0.966 for XGB (Figure S2c), and 0.965 for LGBM (Figure S2e), while the PR-AUC values are 0.925, 0.945, and 0.938 for RF (Figure S2b), XGB (Figure S2d), and LGBM (Figure S2f), respectively. All the models demonstrate excellent classification performance, indicating that the selected compositional descriptors contain sufficient information to distinguish cubic from non-cubic perovskite oxides.

### 3.3. Large-Scale Screening of Unexplored Compositional Space

**Figure 7:**
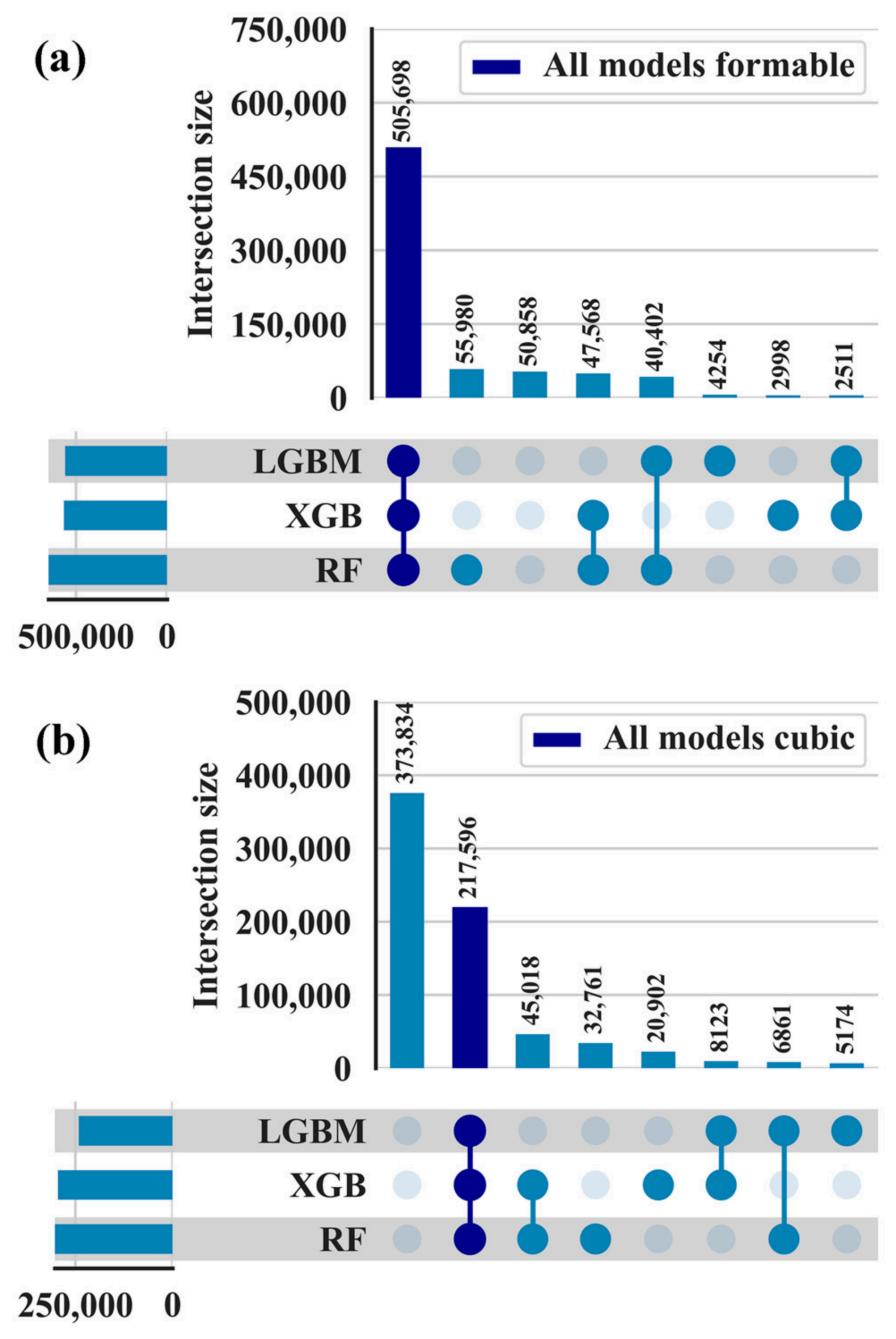
Model agreement analysis for the screening of (**a**) perovskite formability and (**b**) cubic phase using RF, XGB, and LGBM models.

After generating 710,269 virtual ABO_3_ combinations using 7 A-site cations and 14 B-site cations with a compositional step size of 0.1, the finalized RF, XGB, and LGBM models were sequentially applied to predict formable and cubic perovskite oxides. Figure 7 summarizes the agreement and divergence among the three ML models (RF, XGB, and LGBM) in predicting perovskite formability and cubic structure across the combinations. As shown in Figure 7a, a dominant fraction of compositions (505,698) is consistently predicted to be formable by all three models. In contrast, the number of compositions predicted as formable by only one or two models is significantly smaller (e.g., 55,980, 47,568, and 40,402), suggesting that model disagreement is relatively limited. This high level of consistency implies that perovskite formability is governed by well-defined and robust descriptors that can be effectively captured across different ML algorithms. The relatively small subsets of disagreement likely correspond to compositions located near classification boundaries, where minor differences in model structure or feature weighting lead to divergent predictions.

In comparison, the agreement pattern for cubic perovskite prediction in Figure 7b is more consistent. 373,834 compositions are predicted as non-cubic by all three models, while 217,596 combinations are unanimously classified as cubic. Meanwhile, the remaining compositions are distributed across partial overlaps, reflecting a lower degree of model disagreement than in the formability task.

Given that 505,698 compositions are predicted to be formable, and 217,596 combinations are identified as cubic perovskite oxides, 189,247 compositions were simultaneously predicted to be both formable and cubic (Figure S3). Some predicted candidates, such as BaCo_0.1_Ti_0.9_O_3−δ_ [38], SrCo_0.5_Mn_0.5_O_3−δ_ [39], SrMn_0.7_Mo_0.3_O_3−δ_ [40], Ba_0.8_Sr_0.2_Co_0.6_Fe_0.2_Nb_0.2_O_3−δ_ [41], Ba_0.6_Sr_0.4_Co_0.4_Fe_0.6_O_3−δ_ [42], BaCo_0.7_Nb_0.2_Ta_0.1_O_3−δ_ [43], and Ba_0.9_La_0.1_Co_0.7_Fe_0.2_Nb_0.1_O_3−δ_ [44], have been previously synthesized and confirmed experimentally to crystallize in cubic perovskite structures, validating the predictive capability of the proposed models. The top 20 predicted formable and cubic perovskite oxides ranked by prediction probability are shown in Table S5. Interestingly, not all compositions predicted as cubic were simultaneously classified as formable. This inconsistency suggests the intrinsic difference between the two prediction tasks: while formability reflects the geometric feasibility of forming a perovskite phase, cubic prediction primarily captures structural symmetry characteristics. As a result, the cubic model may assign high probability to cubic-like configurations even for compositions that are unlikely to be experimentally realizable as formable perovskite oxides. Therefore, the 189,247 compositions are considered reliable candidates for experimentally accessible cubic perovskite oxides in subsequent analysis. This observation further justifies the necessity of a two-stage screening framework.

Furthermore, the fact that the fully consistent cubic subset is significantly smaller than the fully consistent formable subset implies that achieving cubic symmetry represents a stricter criterion, highlighting the intrinsically greater complexity of cubic phase prediction. Unlike formability, which is largely determined by geometric feasibility, the stabilization of the cubic structure is highly sensitive to subtle structural factors such as lattice distortion, octahedral tilting, and volume mismatch [6,7,9,45].

**Figure 8:**
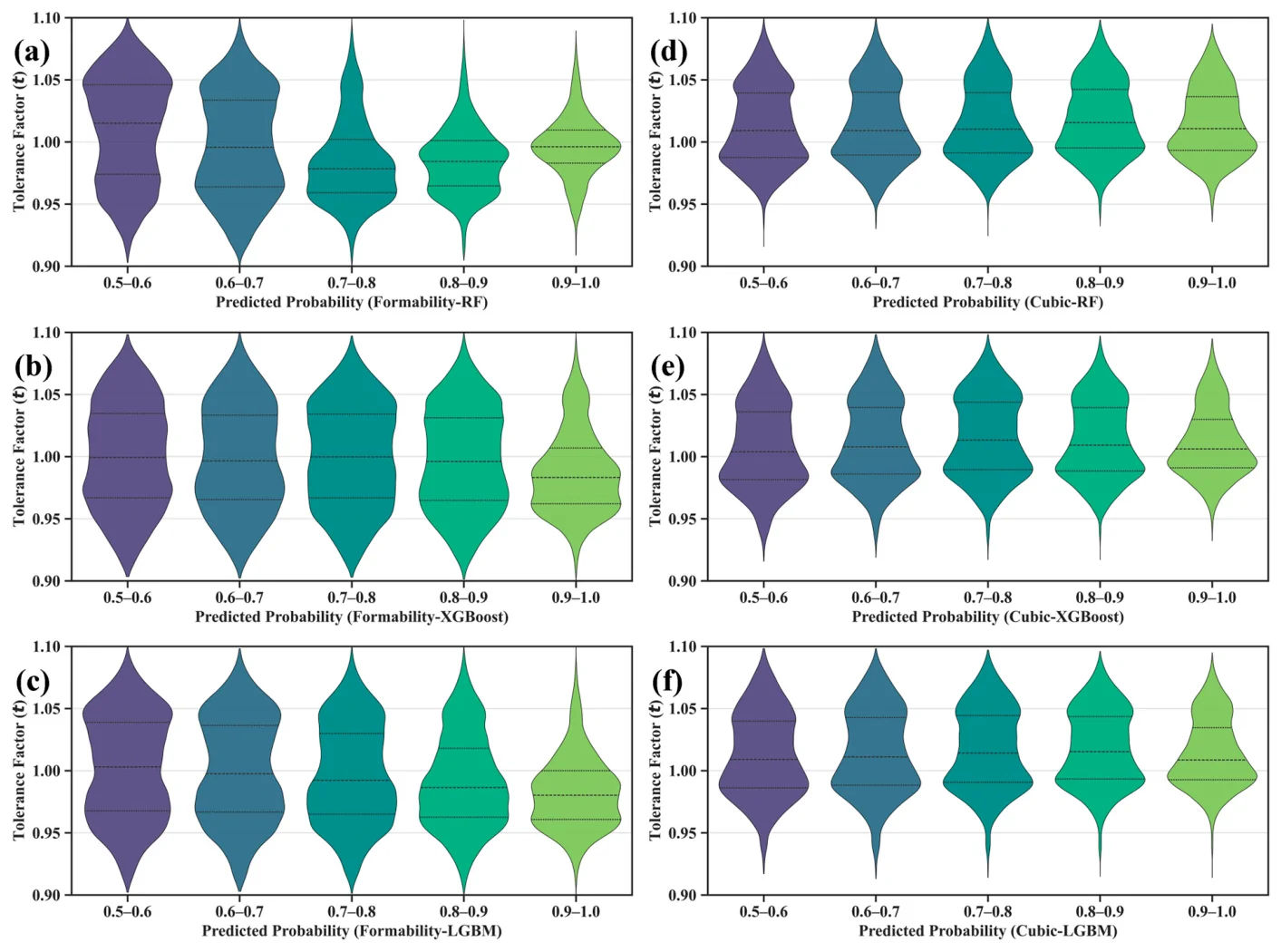
Relationship between *t* and predicted probability for formability (**a**–**c**) and cubic symmetry (**d**–**f**) in perovskite oxides.

The violin plots in Figure 8 depict the distributions of *t* as a function of probability for predicted formable and cubic perovskite oxides, as obtained from the RF, XGB, and LGBM models. For perovskite formability (Figure 8a–c), a clear yet relatively broad trend is observed. As the predicted probability increases from 0.5–0.6 to 0.9–1.0, the distributions of *t* gradually concentrate within a narrower range, primarily around ~0.95–1.00. However, the spread of *t* values remains relatively wide across all probability intervals, indicating that compositions with similar *t* can still exhibit markedly different predicted probabilities. The persistence of substantial overlap among adjacent probability bins further proves that additional chemical and electronic descriptors are required to capture the complexity of perovskite formation in multicomponent systems.

In contrast, a more pronounced and systematic trend is observed for cubic perovskite prediction (Figure 8d–f). As the predicted probability increases, the *t* distributions become progressively narrower and shift toward values closer to unity. In the highest probability interval (0.9–1.0), *t* is tightly clustered around ~1.00, reflecting the well-established geometric requirement for ideal cubic structure. Compared to the formability case, the reduced variance and clearer separation among probability bins indicate that *t* plays a more decisive role in governing cubic symmetry. This behavior is consistent across all three models, demonstrating the robustness of this relationship. However, despite this stronger correlation, some degree of overlap between probability intervals remains even for cubic prediction. This implies that although *t* is a key descriptor, it still cannot fully capture the structural nuances that determine whether a perovskite adopts a cubic phase. The consistency of trends among RF, XGB, and LGBM further demonstrates that the observed relationships are not model-specific but rather reflect intrinsic structure-property correlations within the dataset.

## 4. Conclusions

In this work, a two-stage machine learning framework was developed to efficiently predict perovskite oxide formability and cubic structure in doped ABO_3_ systems. By integrating compositional descriptors with ensemble learning algorithms (RF, XGB, and LGBM), the proposed approach achieved robust and consistent predictive performance. Among the models, XGB exhibited the best overall accuracy. Feature importance analysis and SHAP interpretation confirmed that geometric descriptors, particularly those related to atomic radii, were identified as the dominant factors for formability prediction, while volume-related descriptors played a more critical role in determining the cubic phase. The trained models were subsequently applied to screen 710,269 virtual ABO_3_ compositions, leading to the identification of 189,247 candidate compositions predicted to be both formable and cubic. This high-throughput screening demonstrates the effectiveness of the proposed framework in navigating vast compositional spaces that are otherwise inaccessible to conventional approaches.

## Figures and Tables

**Table 1:** Performance of the final RF, XGB, and LGBM models for predicting perovskite formability.

Model	RF	XGB	LGBM
Accuracy	0.907	0.910	0.904
Precision	0.927	0.940	0.935
Recall	0.944	0.935	0.931
F1 score	0.936	0.937	0.933

**Table 2:** Performance of the final RF, XGB, and LGBM models for predicting cubic perovskite oxides.

Model	RF	XGB	LGBM
Accuracy	0.920	0.924	0.920
Precision	0.861	0.876	0.861
Recall	0.912	0.902	0.912
F1 score	0.886	0.889	0.886

## Data Availability

The original contributions presented in this study are included in the article/Supplementary Material. Further inquiries can be directed to the corresponding authors.

## Supplementary Materials

The following supporting information can be downloaded at: https://www.mdpi.com/article/10.3390/ma19184015/s1, Figure S1: ROC and PR curves of the RF, XGB, and LGBM models for perovskite formability prediction. Figure S2: ROC and PR curves of the RF, XGB, and LGBM models for cubic perovskite oxide prediction. Figure S3: Model agreement analysis for the screening of formable and cubic perovskite oxides using RF, XGB, and LGBM models. Table S1: 1502 ABO_3_ compounds in the input dataset. Table S2: Elemental attributes used to construct the composition-based descriptors in this study. Table S3: Hyperparameters for the final three modes for formability prediction. Table S4: Hyperparameters for the final three modes for cubic prediction. Table S5: Top 20 predicted formable and cubic perovskite oxides ranked by prediction probability. Reference [46] is cited in the supplementary materials.
