# Supplementary material for: Machine Learning-Driven Prediction of Formability and Cubic Phase in Compositionally Complex ABO3 Perovskite Oxides
Source: Materials (Basel). 2026 Sep 21;19(18):4015. doi: 10.3390/ma19184015 (PMC13608706; doi:10.3390/ma19184015)
Supplement: Supplementary file 1 [file materials-19-04015-s001.zip › materials-4534997-supplementary.pdf]

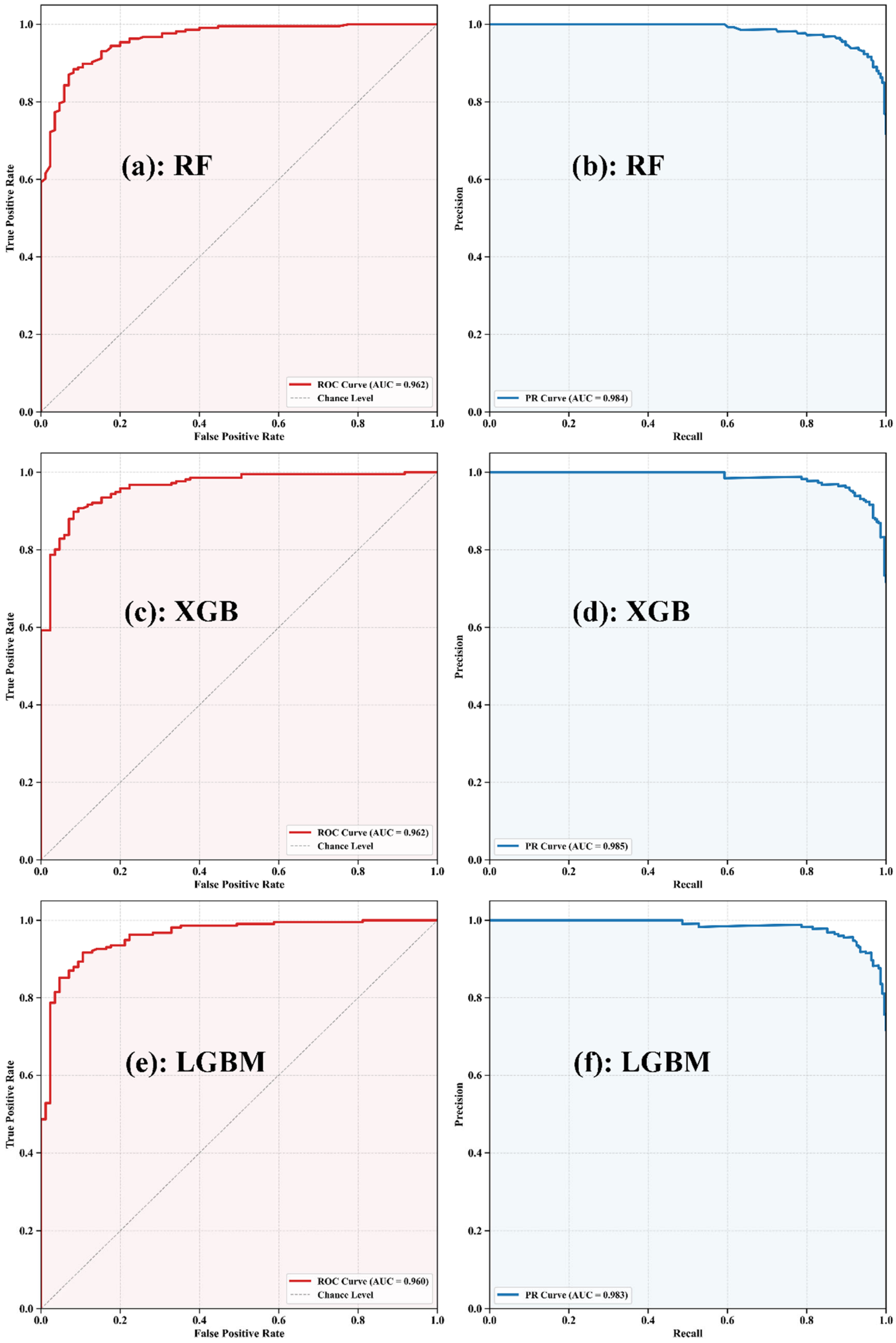

Figure S1. ROC and PR curves of the RF, XGB, and LGBM models for perovskite formability prediction.

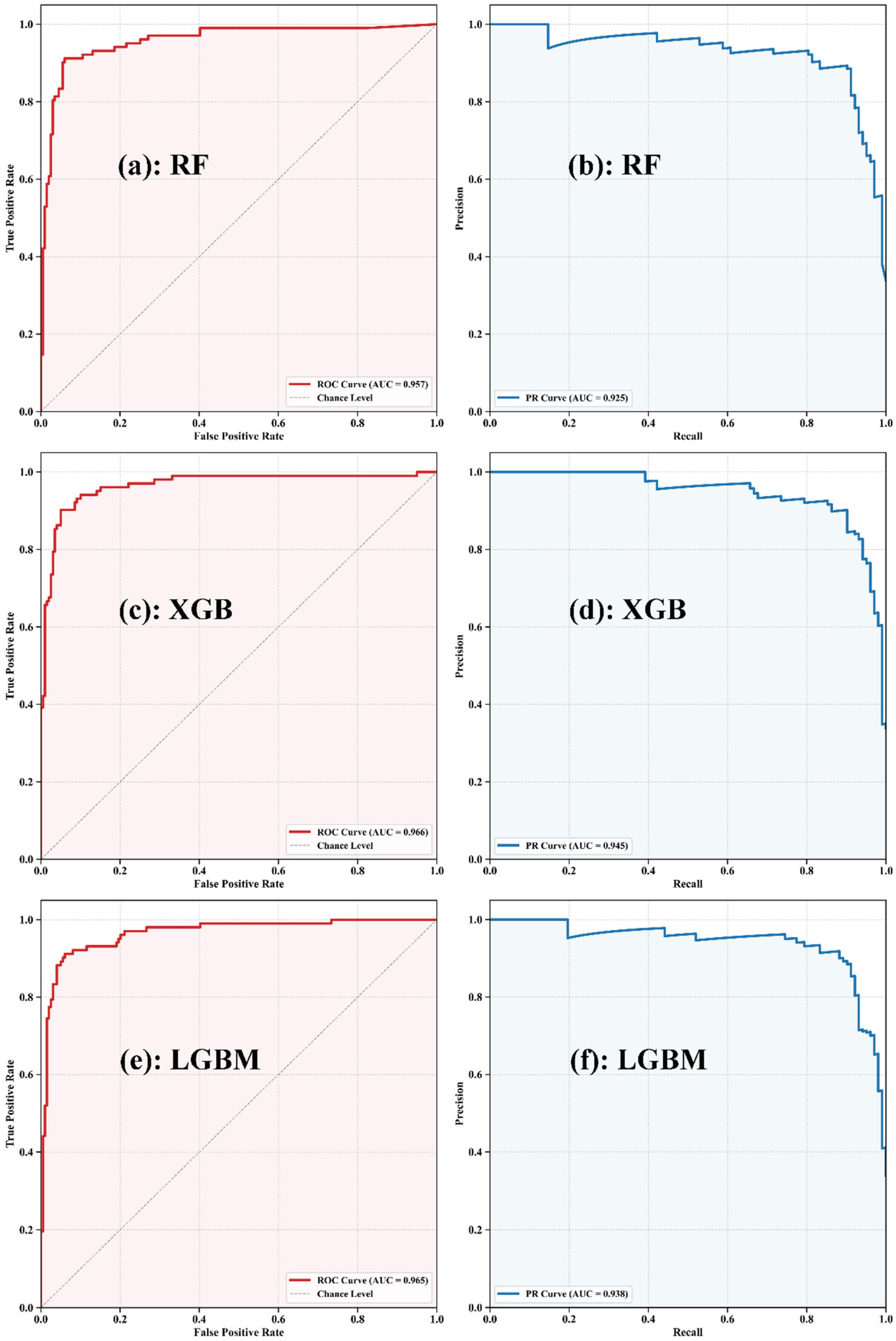

Figure S2. ROC and PR curves of the RF, XGB, and LGBM models for cubic perovskite oxide prediction.

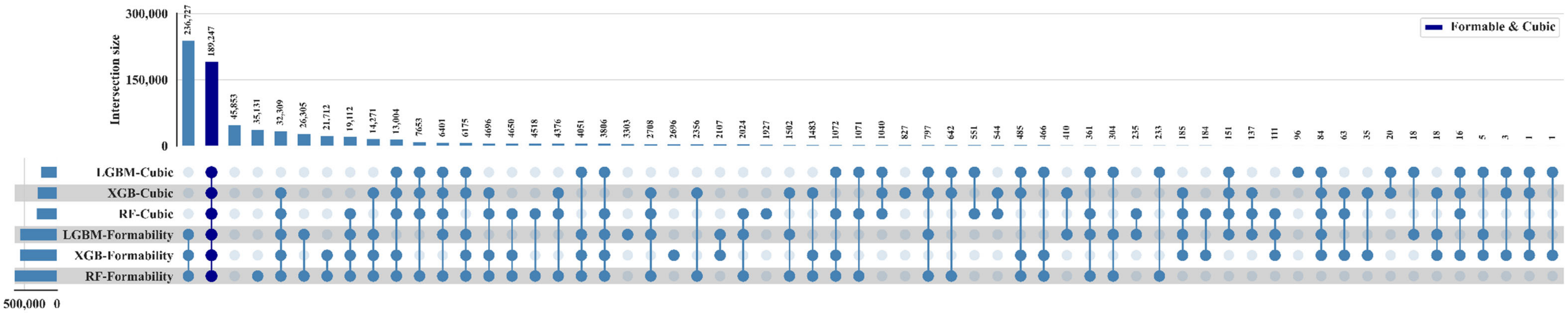

Figure S3. Model agreement analysis for the screening of formable and cubic perovskite oxides using RF, XGB, and LGBM models.

Table S1. 1502 ABO_3 compounds in the input dataset.

**ABO_3 compounds**		
AgBiO3	HgTiO3	Nd0.3Sr0.7Fe0.1Co0.9O3
AgNbO3	HoAlO3	Nd0.3Sr0.7Fe0.3Co0.7O3
AgPO3	HoBO3	Nd0.3Sr0.7Fe0.5Co0.5O3
AgSbO3	HoCo0.5Mn0.5O3	Nd0.3Sr0.7Fe0.7Co0.3O3
AgTaO3	HoCoO3	Nd0.3Sr0.7Fe0.8Cu0.2O3
AgVO3	HoCrO3	Nd0.3Sr0.7Fe0.9Co0.1O3
AlBO3	HoFeO3	Nd0.3Sr0.7FeO3
AlFeO3	HoNi0.2Co0.2Fe0.2Mn0.2Cr0.2O3	Nd0.4Sr0.6Fe0.7Co0.3O3
AsBO3	HoNiO3	Nd0.4Sr0.6Fe0.8Cu0.2O3
Ba0.1Co0.9Al0.1O3	HoRhO3	Nd0.5Ba0.5Fe0.9Co0.1O3
Ba0.1Co0.9Y0.1O3	HoScO3	Nd0.5Ba0.5Fe0.9Cu0.1O3
Ba0.1Sr0.9Co0.9Al0.1O3	HoVO3	Nd0.5Ba0.5Fe0.9Ni0.1O3
Ba0.1Sr0.9Co0.9Y0.1O3	InBO3	Nd0.5Ba0.5FeO3
Ba0.2La0.3Sr0.5Co0.6Fe0.4O3	InCrO3	Nd0.5Sr0.5Fe0.7Co0.3O3
Ba0.2Sr0.8Co0.9Al0.1O3	InFeO3	Nd0.5Sr0.5Fe0.8Cu0.2O3
Ba0.2Sr0.8Co0.9Y0.1O3	InRhO3	Nd0.5Sr0.5Fe0.9Mo0.1O3
Ba0.3Sr0.7Co0.9Al0.1O3	KAsO3	Nd0.5Sr0.5MnO3
Ba0.3Sr0.7Co0.9Y0.1O3	KBiO3	Nd0.65Sr0.2Ca0.15CoO3
Ba0.425Sr0.425Sm0.15Co0.8Fe0.2O3	KNbO3	Nd0.67Ba0.33Mn0.98Fe0.02O3
Ba0.45Sr0.45Ce0.1Co0.8Fe0.2O3	KPO3	Nd0.6Sr0.2Ca0.2CoO3
Ba0.45Sr0.45Sm0.1Co0.8Fe0.2O3	KSbO3	Nd0.6Sr0.4Fe0.7Co0.3O3
Ba0.475Sr0.475Sm0.05Co0.8Fe0.2O3	KTaO3	Nd0.6Sr0.4Fe0.8Cu0.2O3
Ba0.4Sr0.4Ce0.2Co0.8Fe0.2O3	KVO3	Nd0.6Sr0.4FeO3
Ba0.4Sr0.6Co0.9Al0.1O3	La0.125Sr0.875Co0.875Ta0.125O3	Nd0.6Sr0.4Mn0.85Ni0.15O3

Ba0.4Sr0.6Co0.9Y0.1O3	La0.18Sr0.12Ba0.7Co0.55Fe0.38Nb0.07O3	Nd0.6Sr0.4Mn0.8Ni0.2O3
Ba0.5La0.5FeO3	La0.1Ba0.9Co0.4Fe0.6O3	Nd0.6Sr0.4Mn0.95Ni0.05O3
Ba0.5Sm0.5Co0.8Zr0.2O3	La0.1Ba0.9Co0.5Fe0.5O3	Nd0.6Sr0.4Mn0.9Ni0.1O3
Ba0.5Sm0.5Fe0.8Zr0.2O3	La0.1Ba0.9Co0.6Fe0.4O3	Nd0.6Sr0.4MnO3
Ba0.5Sr0.5CeO3	La0.1Ba0.9Co0.7Fe0.3O3	Nd0.75Ba0.25Mn0.5Fe0.5O3
Ba0.5Sr0.5Co0.05Zn0.15Fe0.8O3	La0.1Ba0.9Co0.8Fe0.2O3	Nd0.75Ca0.25Mn0.5Fe0.5O3
Ba0.5Sr0.5Co0.10Zn0.10Fe0.8O3	La0.1Ba0.9Co0.9Fe0.1O3	Nd0.75Sr0.25Mn0.5Fe0.5O3
Ba0.5Sr0.5Co0.15Zn0.05Fe0.8O3	La0.1Bi0.7Sr0.2FeO3	Nd0.75Sr0.2Ca0.05CoO3
Ba0.5Sr0.5Co0.1Fe0.9O3	La0.1Ca0.9Cr0.2Ti0.8O3	Nd0.7Ba0.3MnO3
Ba0.5Sr0.5Co0.1Zn0.1Fe0.8O3	La0.1Ca0.9Cr0.5Ti0.5O3	Nd0.7Ca0.15Sr0.15MnO3
Ba0.5Sr0.5Co0.2Fe0.8O3	La0.1Ca0.9Fe0.8Ni0.2O3	Nd0.7Ca0.3MnO3
Ba0.5Sr0.5Co0.3Fe0.7O3	La0.1Ca0.9Mn0.85Ge0.15O3	Nd0.7Sr0.2Ca0.1CoO3
Ba0.5Sr0.5Co0.4Fe0.6O3	La0.1Sr0.9Co0.2Fe0.6Zr0.2O3	Nd0.7Sr0.3Fe0.7Co0.3O3
Ba0.5Sr0.5Co0.5Fe0.5O3	La0.1Sr0.9Co0.7Fe0.3O3	Nd0.7Sr0.3Fe0.8Cu0.2O3
Ba0.5Sr0.5Co0.6Fe0.4O3	La0.1Sr0.9Co0.7Mn0.3O3	Nd0.7Sr0.3FeO3
Ba0.5Sr0.5Co0.76Fe0.19P0.05O3	La0.1Sr0.9Co0.85Nb0.15O3	Nd0.7Sr0.3MnO3
Ba0.5Sr0.5Co0.7Fe0.28Ce0.02O3	La0.1Sr0.9Co0.8Fe0.2O3	Nd0.85Sr0.15MnO3
Ba0.5Sr0.5Co0.7Fe0.28La0.02O3	La0.1Sr0.9Co0.8Mn0.2O3	Nd0.8Sr0.2CoO3
Ba0.5Sr0.5Co0.7Fe0.28Pr0.02O3	La0.1Sr0.9Co0.8Nb0.2O3	Nd0.8Sr0.2FeO3
Ba0.5Sr0.5Co0.7Fe0.3O3	La0.1Sr0.9Co0.9Fe0.1O3	Nd0.9Sr0.1Fe0.7Co0.3O3
Ba0.5Sr0.5Co0.8Fe0.1Ni0.1O3	La0.1Sr0.9Co0.9Mn0.1O3	Nd0.9Sr0.1FeO3
Ba0.5Sr0.5Co0.8Fe0.2O3	La0.1Sr0.9Co0.9Nb0.1O3	NdAlO3
Ba0.5Sr0.5Co0.8Zr0.2O3	La0.1Sr0.9CoO3	NdBO3

Ba0.5Sr0.5Co0.9Al0.1O3	La0.24Sr0.16Ba0.6Co0.5Fe0.44Nb0.06O3	NdCo0.5Mn0.5O3
Ba0.5Sr0.5Co0.9Fe0.1O3	La0.25Pr0.75Co0.5Ni0.5O3	NdCo0.95Ni0.05O3
Ba0.5Sr0.5Co0.9Y0.1O3	La0.25Sr0.75Co0.5Mn0.5O3	NdCoO3
Ba0.5Sr0.5Cu0.2Fe0.8O3	La0.2Ba0.8Co0.4Fe0.6O3	NdCrO3
Ba0.5Sr0.5Fe0.6Ce0.4O3	La0.2Ba0.8Co0.5Fe0.5O3	NdDyO3
Ba0.5Sr0.5Fe0.8Cu0.1Ti0.1O3	La0.2Ba0.8Co0.6Fe0.4O3	NdFe0.7Co0.3O3
Ba0.5Sr0.5Fe0.8Cu0.2O3	La0.2Ba0.8Co0.7Fe0.3O3	NdFeO3
Ba0.5Sr0.5Fe0.8Zr0.2O3	La0.2Ba0.8Co0.8Fe0.2O3	NdGaO3
Ba0.5Sr0.5Fe0.95Sb0.05O3	La0.2Ba0.8Co0.9Fe0.1O3	NdInO3
Ba0.5Sr0.5Fe0.9Cu0.1O3	La0.2Bi0.6Sr0.2FeO3	NdMn0.5Fe0.5O3
Ba0.5Sr0.5Fe0.9Mo0.1O3	La0.2Ca0.8Cr0.2Ti0.8O3	NdMnO3
Ba0.5Sr0.5Fe0.9Nb0.1O3	La0.2Ca0.8Cr0.5Ti0.5O3	NdNiO3
Ba0.5Sr0.5Fe0.9Sb0.1O3	La0.2Ca0.8Fe0.8Ni0.2O3	NdRhO3
Ba0.5Sr0.5FeO3	La0.2Nd0.2Ca0.2Sr0.2Ba0.2MnO3	NdScO3
Ba0.5Sr0.5Zn0.2Fe0.8O3	La0.2Nd0.2Gd0.2Sr0.2Ba0.2Co0.2Fe0.8O3	NdTiO3
Ba0.6La0.4Fe0.8Mo0.1Ni0.1O3	La0.2Nd0.2Pr0.2Sr0.2Ba0.2MnO3	NdVO3
Ba0.6La0.4FeO3	La0.2Nd0.2Sm0.2Ca0.2Sr0.2MnO3	NdYbO3
Ba0.6Sr0.4Co0.9Al0.1O3	La0.2Nd0.2Sm0.2Y0.2Gd0.2MnO3	Ni0.25Mn0.25Zn0.25Mg0.25TiO3
Ba0.6Sr0.4Co0.9Y0.1O3	La0.2Pr0.2Ba0.2Sr0.2Ca0.2CoO3	Ni0.333Zn0.333Mg0.334TiO3
Ba0.75La0.25InO3	La0.2Pr0.2Ca0.2Sr0.2Ba0.2MnO3	Ni0.5Mg0.5TiO3
Ba0.75Sr0.25Fe0.875Ga0.125O3	La0.2Pr0.2Eu0.2Ce0.2Sr0.2FeO3	Ni0.5Mn0.5TiO3
Ba0.75Sr0.25Fe0.875Y0.125O3	La0.2Pr0.2Nd0.2Ba0.2Sr0.2CoO3	NiIn0.5Sb0.5O3
Ba0.7La0.3FeO3	La0.2Pr0.2Nd0.2Sm0.2Ba0.1Sr0.1Co0.2Fe0.6Ni0.1Cu0.1O3	NiMnO3
Ba0.7Nd0.3Co0.2Ti0.8O3	La0.2Pr0.2Nd0.2Sm0.2Ba0.2CoO3	NiTiO3
Ba0.7Sr0.3Co0.9Al0.1O3	La0.2Pr0.2Nd0.2Sm0.2Sr0.2Co0.5Fe0.5O3	PbCeO3
Ba0.7Sr0.3Co0.9Y0.1O3	La0.2Pr0.2Nd0.2Sm0.2Sr0.2CoO3	PbCO3
Ba0.85Ca0.15Zr0.1Ti0.9O3	La0.2Pr0.2Nd0.2Sm0.2Sr0.2FeO3	PbCrO3

Ba0.875La0.125In0.875Sn0.125O3	La0.2Pr0.2Nd0.2Sm0.2Sr0.2MnO3	PbGeO3
Ba0.875La0.125In0.875Zr0.125O3	La0.2Sr0.8Co0.2Fe0.6Zr0.2O3	PbHfO3
Ba0.8La0.2FeO3	La0.2Sr0.8Co0.2Fe0.8O3	PbSc0.37Nb0.37Ti0.26O3
Ba0.8Nd0.2Co0.2Ti0.8O3	La0.2Sr0.8Co0.7Fe0.3O3	PbSiO3
Ba0.8Sr0.2Co0.9Al0.1O3	La0.2Sr0.8Co0.7Mn0.3O3	PbSnO3
Ba0.8Sr0.2Co0.9Y0.1O3	La0.2Sr0.8Co0.8Fe0.2O3	PbTiO3
Ba0.93Bi0.07Ti0.93Sc0.07O3	La0.2Sr0.8Co0.8Mn0.2O3	PbVO3
Ba0.94Sr0.06Fe0.8Al0.05Mg0.15O3	La0.2Sr0.8Co0.9Fe0.1O3	PbZn0.39Nb0.61O3
Ba0.94Sr0.06Fe0.8Al0.1Mg0.1O3	La0.2Sr0.8Co0.9Mn0.1O3	PbZr0.55Ti0.45O3
Ba0.94Sr0.06Fe0.8Al0.2O3	La0.2Sr0.8Cu0.4Co0.6O3	PbZr0.58Ti0.42O3
Ba0.94Sr0.06Fe0.8Mg0.2O3	La0.33Ba0.47Gd0.2Co0.7Fe0.3O3	PbZr0.6Ti0.4O3
Ba0.95Ca0.05Co0.1Fe0.9O3	La0.33Ba0.57Gd0.1Co0.7Fe0.3O3	PbZrO3
Ba0.95Ca0.05Co0.2Fe0.8O3	La0.33Ba0.62Gd0.05Co0.7Fe0.3O3	Pr0.1Ba0.9Co0.7Fe0.3O3
Ba0.95Ca0.05Co0.4Fe0.6O3	La0.33Ba0.67Co0.7Fe0.3O3	Pr0.2Ce0.8Co0.5Mn0.5O3
Ba0.95Ca0.05Co0.6Fe0.4O3	La0.35Ca0.15Ba0.5FeO3	Pr0.2Sr0.2La0.2Ba0.2Ca0.2Co0.2Fe0.8O3
Ba0.95Ca0.05Co0.8Fe0.2O3	La0.36Sr0.24Ba0.4Co0.4Fe0.56Nb0.04O3	Pr0.2Sr0.2La0.2Ba0.2Nd0.2Co0.2Fe0.8O3
Ba0.95Ca0.05Co0.9Fe0.1O3	La0.3Ba0.7Co0.4Fe0.6O3	Pr0.4Ce0.6Co0.5Mn0.5O3
Ba0.95Ca0.05CoO3	La0.3Ba0.7Co0.5Fe0.5O3	Pr0.4Sr0.6Co0.16Fe0.64Mo0.2O3
Ba0.95Ca0.05Fe0.95In0.05O3	La0.3Ba0.7Co0.6Fe0.4O3	Pr0.4Sr0.6Co0.18Fe0.72Mo0.1O3
Ba0.95Ca0.05FeO3	La0.3Ba0.7Co0.7Fe0.3O3	Pr0.4Sr0.6Co0.19Fe0.76Mo0.05O3
Ba0.95Gd0.05FeO3	La0.3Ba0.7Co0.8Fe0.2O3	Pr0.4Sr0.6Co0.2Fe0.8O3
Ba0.95La0.05Fe0.85Cu0.15O3	La0.3Ba0.7Co0.9Fe0.1O3	Pr0.4Sr0.6Fe0.875Mo0.125O3
Ba0.95La0.05Fe0.8Zn0.2O3	La0.3Bi0.5Sr0.2FeO3	Pr0.54Er0.06Ca0.1Sr0.3MnO3

Ba0.95La0.05Fe0.95P0.05O3	La0.3Ca0.2Ba0.5FeO3	Pr0.56Er0.04Ca0.1Sr0.3MnO3
Ba0.95La0.05Fe0.9Nb0.1O3	La0.3Ca0.7Cr0.2Ti0.8O3	Pr0.58Er0.02Ca0.1Sr0.3MnO3
Ba0.95La0.05FeO3	La0.3Ca0.7Cr0.5Ti0.5O3	Pr0.5Ba0.1Ca0.4FeO3
Ba0.95Nd0.05FeO3	La0.3Ca0.7Fe0.7Cr0.3O3	Pr0.5Ba0.5Co0.7Fe0.225W0.075O3
Ba0.95Sm0.05FeO3	La0.3Ca0.7Fe0.8Cr0.2O3	Pr0.5Ba0.5Co0.7Fe0.25W0.05O3
Ba0.97Nd0.03FeO3	La0.3Ca0.7Fe0.8Ni0.2O3	Pr0.5Ba0.5Co0.7Fe0.275W0.025O3
Ba0.9La0.1Co0.7Fe0.2Nb0.1O3	La0.3Ca0.7Fe0.9Cr0.1O3	Pr0.5Ba0.5Co0.7Fe0.2W0.1O3
Ba0.9La0.1Co0.7Fe0.3O3	La0.3Sr0.2Ba0.5Co0.45Fe0.5Nb0.05O3	Pr0.5Ba0.5Co0.7Fe0.3O3
Ba0.9La0.1FeO3	La0.3Sr0.7Co0.2Fe0.6Zr0.2O3	Pr0.5Ba0.5Fe0.8Ni0.2O3
Ba0.9Nd0.1Co0.2Ti0.8O3	La0.3Sr0.7Co0.7Fe0.3O3	Pr0.5Ba0.5FeO3
Ba0.9Nd0.1FeO3	La0.3Sr0.7Co0.7Mn0.3O3	Pr0.5Ca0.5FeO3
Ba0.9Sr0.1Co0.9Al0.1O3	La0.3Sr0.7Co0.8Mn0.2O3	Pr0.5Sr0.5Co0.2Fe0.8O3
Ba0.9Sr0.1Co0.9Bi0.1O3	La0.3Sr0.7Co0.9Fe0.1O3	Pr0.5Sr0.5CoO3
Ba0.9Sr0.1Co0.9Cr0.1O3	La0.3Sr0.7Co0.9Mn0.1O3	Pr0.5Sr0.5Cr0.1Fe0.7Ni0.2O3
Ba0.9Sr0.1Co0.9Ga0.1O3	La0.3Sr0.7Fe0.5Al0.3Cr0.2O3	Pr0.5Sr0.5Cr0.1Fe0.8Ni0.1O3
Ba0.9Sr0.1Co0.9In0.1O3	La0.3Sr0.7Fe0.5Ga0.5O3	Pr0.5Sr0.5Fe0.6Cu0.4O3
Ba0.9Sr0.1Co0.9Y0.1O3	La0.3Sr0.7Fe0.6Al0.3Cr0.1O3	Pr0.5Sr0.5Fe0.8Cu0.2O3
Ba0.9Sr0.1CoO3	La0.3Sr0.7Fe0.6Al0.4O3	Pr0.5Sr0.5Fe0.9Mo0.1O3
BaBi0.05Co0.75Nb0.2O3	La0.3Sr0.7Fe0.6Ga0.4O3	Pr0.5Sr0.5MnO3
BaBi0.05Co0.85Nb0.1O3	La0.3Sr0.7Fe0.7Ga0.3O3	Pr0.6Ca0.1Sr0.3Mn0.925Fe0.075O3
BaBi0.05Co0.8Nb0.15O3	La0.3Sr0.7Fe0.8Al0.2O3	Pr0.6Ca0.1Sr0.3MnO3
BaBi0.05Co0.8Ta0.15O3	La0.3Sr0.7Fe0.8Ga0.2O3	Pr0.6Ca0.4FeO3
BaBi0.05Co0.95O3	La0.3Sr0.7Fe0.9Ga0.1O3	Pr0.6Ce0.4Co0.5Mn0.5O3
BaBi0.05Co0.9Nb0.05O3	La0.3Sr0.7FeO3	Pr0.6Sr0.4Co0.2Fe0.8O3
BaBi0.05Zr0.1Co0.85O3	La0.45Ca0.05Ba0.5FeO3	Pr0.6Sr0.4CoO3
BaBi0.05Zr0.1Co0.8Nb0.05O3	La0.4Ba0.1Sr0.5Co0.8Ta0.2O3	Pr0.6Sr0.4Fe0.7Ni0.2Mo0.1O3
BaBi0.1Co0.8Nb0.1O3	La0.4Ba0.2Sr0.4Co0.8Ta0.2O3	Pr0.6Sr0.4Fe0.8Cu0.1W0.1O3

BaCe0.05Fe0.95O3
BaCe0.05Mn0.95O3
BaCe0.08Y0.02Fe0.9O3
BaCe0.15Mn0.85O3
BaCe0.16Y0.04Fe0.8O3
BaCe0.1Zr0.2Y0.1Fe0.6O3
BaCe0.24Y0.06Fe0.7O3
BaCe0.25Mn0.75O3
BaCe0.2Fe0.5Pr0.3O3
BaCe0.2Fe0.6Pr0.2O3
BaCe0.2Fe0.7Pr0.1O3
BaCe0.2Fe0.8O3
BaCe0.2Zr0.2Y0.1Fe0.5O3
BaCe0.3Fe0.7O3
BaCe0.3Y0.2Fe0.3Co0.2O3
BaCe0.3Zr0.2Y0.1Fe0.4O3
BaCe0.4Fe0.4Co0.2O3
BaCe0.4Fe0.6O3
BaCe0.4Zr0.2Y0.1Fe0.3O3
BaCe0.5Co0.5O3
BaCe0.5Fe0.2Bi0.3O3
BaCe0.5Fe0.3Bi0.2O3
BaCe0.5Fe0.4Bi0.1O3
BaCe0.5Fe0.5O3
BaCe0.5Mn0.5O3
BaCe0.5Zr0.2Y0.1Fe0.2O3
BaCe0.6Fe0.4O3
BaCe0.6Zr0.2Y0.1Fe0.1O3
BaCe0.75Mn0.25O3
BaCe0.7Fe0.3O3
BaCe0.7Zr0.2Y0.1O3
BaCe0.8Fe0.1Y0.1O3

La0.4Ba0.6CoO3
La0.4Bi0.2Sr0.4Co0.2Fe0.8O3
La0.4Bi0.4Sr0.2FeO3
La0.4Ca0.1Ba0.5FeO3
La0.4Ca0.6Cr0.2Ti0.8O3
La0.4Ca0.6Cr0.5Ti0.5O3
La0.4Ca0.6Fe0.8Ni0.2O3
La0.4Sr0.6Co0.2Fe0.6Zr0.2O3
La0.4Sr0.6Co0.2Fe0.7Nb0.1O3
La0.4Sr0.6Co0.4Fe0.6O3
La0.4Sr0.6Co0.6Fe0.4O3
La0.4Sr0.6Co0.6Mn0.4O3
La0.4Sr0.6Co0.7Fe0.3O3
La0.4Sr0.6Co0.8Fe0.2O3
La0.4Sr0.6Co0.8Ta0.2O3
La0.4Sr0.6Co0.9Fe0.1O3
La0.4Sr0.6Co0.9Ta0.1O3
La0.4Sr0.6CoO3
La0.55Eu0.15Ba0.3MnO3
La0.5Ba0.5CoO3
La0.5Ba0.5Fe0.95Mo0.05O3
La0.5Ba0.5FeO3
La0.5Bi0.1Sr0.4Co0.2Fe0.8O3
La0.5Bi0.3Sr0.2FeO3
La0.5Ca0.5Cr0.2Ti0.8O3
La0.5Ca0.5Cr0.5Ti0.5O3
La0.5Ca0.5Fe0.8Ni0.2O3
La0.5Ca0.5Mn0.95Fe0.05O3
La0.5Ca0.5Mn0.95V0.05O3
La0.5Ca0.5Mn0.9V0.1O3
La0.5Na0.5MnO3
La0.5Pr0.5Co0.5Ni0.5O3

Pr0.6Sr0.4Fe0.8Cu0.2O3
Pr0.6Sr0.4Fe0.8Ni0.2O3
Pr0.6Sr0.4FeO3
Pr0.6Sr0.4MnO3
Pr0.7Ba0.3Co0.5Fe0.2Ni0.3O3
Pr0.7Ba0.3Co0.6Fe0.2Ni0.2O3
Pr0.7Ba0.3Co0.7Fe0.2Ni0.1O3
Pr0.7Ba0.3Co0.8Fe0.2O3
Pr0.7Ca0.3FeO3
Pr0.7Ca0.3Mn0.95Co0.05O3
Pr0.7Ca0.3Mn0.9Co0.1O3
Pr0.7Ca0.3MnO3
Pr0.7Sr0.3Co0.2Fe0.8O3
Pr0.7Sr0.3Co0.5Fe0.5O3
Pr0.7Sr0.3CoO3
Pr0.7Sr0.3Fe0.5Ni0.5O3
Pr0.7Sr0.3Fe0.6Ni0.4O3
Pr0.7Sr0.3Fe0.7Ni0.3O3
Pr0.7Sr0.3Fe0.8Ni0.2O3
Pr0.7Sr0.3MnO3
Pr0.85Sr0.15CoO3
Pr0.85Sr0.15MnO3
Pr0.8Ca0.2Fe0.8Co0.2O3
Pr0.8Ca0.2FeO3
Pr0.8Ce0.2Co0.5Mn0.5O3
Pr0.8Na0.05K0.15MnO3
Pr0.8Na0.15K0.05MnO3
Pr0.8Na0.1K0.1MnO3
Pr0.8Na0.2MnO3
Pr0.8Sr0.2Co0.2Fe0.8O3
Pr0.8Sr0.2Fe0.7Ni0.3O3
Pr0.8Sr0.2FeO3

BaCe0.8Fe0.2O3	La0.5Sr0.5Co0.25Fe0.25Mn0.25Ni0.25O3	Pr0.9Ca0.1FeO3
BaCeO3	La0.5Sr0.5Co0.2Fe0.6Zr0.2O3	Pr0.9Sr0.1Co0.2Fe0.8O3
BaCo0.15Fe0.75Hf0.1O3	La0.5Sr0.5Co0.5Fe0.5O3	PrAlO3
BaCo0.1Fe0.8Hf0.1O3	La0.5Sr0.5Co0.5Mn0.5O3	PrBO3
BaCo0.2Fe0.2Zr0.2Sn0.2Pr0.2O3	La0.5Sr0.5Co0.8Cu0.125Nb0.075O3	PrCo0.2Fe0.8O3
BaCo0.2Fe0.5Zr0.3O3	La0.5Sr0.5Co0.8Cu0.15Nb0.05O3	PrCo0.5Mn0.5O3
BaCo0.2Fe0.7Hf0.1O3	La0.5Sr0.5Co0.8Cu0.175Nb0.025O3	PrCo0.5Ni0.5O3
BaCo0.2Zn0.2Ga0.2Zr0.2Y0.2O3	La0.5Sr0.5Co0.8Cu0.2O3	PrCo0.95Ni0.05O3
BaCo0.3Fe0.4Zr0.3O3	La0.5Sr0.5CoO3	PrCoO3
BaCo0.4Fe0.3Zr0.3O3	La0.5Sr0.5Fe0.8Cu0.15Nb0.05O3	PrCrO3
BaCo0.4Fe0.4Zn0.1Y0.1O3	La0.5Sr0.5Fe0.8Cu0.2O3	PrDyO3
BaCo0.4Fe0.4Zn0.2O3	La0.5Sr0.5Fe0.9Mo0.1O3	PrErO3
BaCo0.4Fe0.4Zr0.1Y0.1O3	La0.5Sr0.5Fe0.9Nb0.1O3	PrFeO3
BaCo0.4Fe0.4Zr0.2O3	La0.5Sr0.5Fe0.9W0.1O3	PrGaO3
BaCo0.5Fe0.2Zr0.3O3	La0.5Sr0.5FeO3	PrHoO3
BaCo0.6Fe0.2Sn0.1Y0.1O3	La0.5Sr0.5Mn0.5Cr0.5O3	PrMnO3
BaCo0.6Fe0.2Sn0.2O3	La0.65Ca0.35FeO3	PrNi0.6Co0.4O3
BaCo0.6Fe0.2Y0.1Nb0.1O3	La0.65Eu0.05Ba0.3MnO3	PrNiO3
BaCo0.6Fe0.2Y0.2O3	La0.667Ca0.333Mn0.7In0.3O3	PrRhO3
BaCo0.6Fe0.3Sn0.1O3	La0.667Ca0.333Mn0.85In0.15O3	PrRuO3
BaCo0.7Fe0.15Yb0.15O3	La0.667Ca0.333Mn0.9In0.1O3	PrScO3
BaCo0.7Fe0.25Yb0.05O3	La0.67Ba0.22Sr0.11CoO3	PrTiO3
BaCo0.7Fe0.2Nb0.1O3	La0.67Ba0.22Sr0.11Mn0.7Co0.3O3	PrTmO3
BaCo0.7Fe0.2Ta0.1O3	La0.67Ba0.22Sr0.11Mn0.8Co0.2O3	PrVO3
BaCo0.7Fe0.2Y0.1O3	La0.67Ba0.22Sr0.11Mn0.9Co0.1O3	PrYbO3
BaCo0.7Fe0.2Yb0.1O3	La0.67Ba0.22Sr0.11MnO3	RbNbO3
BaCo0.7Fe0.3O3	La0.6Ba0.4Fe0.8Ni0.2O3	RbPO3
BaCo0.8Zr0.1Y0.1O3	La0.6Ba0.4Ni0.2Fe0.5Ti0.3O3	RbTaO3
BaCO3	La0.6Ba0.4Ni0.2Fe0.6Ti0.2O3	RbVO3
BaCoO3	La0.6Ba0.4Ni0.2Fe0.7Ti0.1O3	Sc0.5Ca0.5MnO3

BaCrO3	La0.6Ba0.4Ni0.2Fe0.8O3	ScAlO3
BaFe0.125Co0.125Zr0.75O3	La0.6Bi0.2Sr0.2FeO3	ScBO3
BaFe0.3Sn0.2Bi0.5O3	La0.6Ca0.35Sr0.05MnO3	ScCrO3
BaFe0.4Sn0.2Bi0.4O3	La0.6Ca0.4Co0.1Mn0.9O3	Sm0.125Sr0.875Co0.875Ta0.125O3
BaFe0.5Ta0.5O3	La0.6Ca0.4Co0.3Mn0.7O3	Sm0.15Pr0.4Sr0.45MnO3
BaFe0.6Bi0.4O3	La0.6Ca0.4CoO3	Sm0.1Sr0.9CoO3
BaFe0.6Ce0.1Co0.3O3	La0.6Ca0.4Cr0.2Ti0.8O3	Sm0.25Pr0.3Sr0.45MnO3
BaFe0.6Co0.3Ce0.1O3	La0.6Ca0.4Cr0.5Ti0.5O3	Sm0.2Sr0.8CoO3
BaFe0.75Cu0.25O3	La0.6Ca0.4Fe0.8Ni0.2O3	Sm0.35Pr0.2Sr0.45MnO3
BaFe0.75Ni0.25O3	La0.6Ca0.4Mn0.1Co0.9O3	Sm0.3Sr0.7CoO3
BaFe0.75Zr0.1Y0.1Cu0.05O3	La0.6Ca0.4Mn0.2Co0.8O3	Sm0.45Pr0.1Sr0.45MnO3
BaFe0.7Bi0.3O3	La0.6Ca0.4Mn0.3Co0.7O3	Sm0.4Sr0.6CoO3
BaFe0.7Co0.2Nb0.1O3	La0.6Ca0.4Mn0.4Co0.6O3	Sm0.5Sr0.45K0.05FeO3
BaFe0.7Zr0.1Y0.1Cu0.1O3	La0.6Ca0.4Mn0.5Co0.5O3	Sm0.5Sr0.5Co0.1Fe0.9O3
BaFe0.85Cu0.15O3	La0.6Ca0.4Mn0.6Co0.4O3	Sm0.5Sr0.5Co0.2Fe0.8O3
BaFe0.85Pr0.15O3	La0.6Ca0.4Mn0.7Co0.3O3	Sm0.5Sr0.5Co0.3Fe0.7O3
BaFe0.85Y0.15O3	La0.6Ca0.4Mn0.8Co0.2O3	Sm0.5Sr0.5Co0.4Fe0.6O3
BaFe0.8Bi0.2O3	La0.6Ca0.4Mn0.9Co0.1O3	Sm0.5Sr0.5Co0.5Fe0.5O3
BaFe0.8Ce0.1Y0.1O3	La0.6Ca0.4MnO3	Sm0.5Sr0.5Co0.6Fe0.4O3
BaFe0.8Cu0.2O3	La0.6Eu0.1Ba0.3MnO3	Sm0.5Sr0.5Co0.7Fe0.3O3
BaFe0.8Pr0.2O3	La0.6Na0.4MnO3	Sm0.5Sr0.5Co0.8Fe0.2O3
BaFe0.8Sn0.2O3	La0.6Nd0.1Sr0.3MnO3	Sm0.5Sr0.5Co0.95Nb0.05O3
BaFe0.8Zr0.1Y0.1O3	La0.6Sr0.4Co0.25Fe0.25Mn0.25Ni0.25O3	Sm0.5Sr0.5Co0.9Fe0.1O3
BaFe0.95Ce0.05O3	La0.6Sr0.4Co0.2Fe0.6Cu0.2O3	Sm0.5Sr0.5Co0.9Nb0.1O3
BaFe0.95In0.05O3	La0.6Sr0.4Co0.2Fe0.6Zr0.2O3	Sm0.5Sr0.5CoO3
BaFe0.95Nb0.05O3	La0.6Sr0.4Co0.2Fe0.7Cu0.1O3	Sm0.5Sr0.5Fe0.8Cu0.2O3
BaFe0.95Pr0.05O3	La0.6Sr0.4Co0.2Fe0.7Pd0.1O3	Sm0.5Sr0.5FeO3
BaFe0.95Sn0.05O3	La0.6Sr0.4Co0.2Fe0.8O3	Sm0.6Sr0.4CoO3
BaFe0.95Y0.05O3	La0.6Sr0.4Co0.3Fe0.4Ni0.30O3	Sm0.7Sr0.3CoO3
BaFe0.95Zr0.05O3	La0.6Sr0.4Co0.3Fe0.55Ni0.15O3	Sm0.8Sr0.2CoO3

BaFe0.9Bi0.1O3	La0.6Sr0.4Co0.3Fe0.7O3	Sm0.8Sr0.2Fe0.8Ti0.15Ru0.05O3
BaFe0.9Cu0.1O3	La0.6Sr0.4Co0.4Fe0.6O3	Sm0.8Sr0.2FeO3
BaFe0.9Hf0.1O3	La0.6Sr0.4CoO3	Sm0.9Sr0.1CoO3
BaFe0.9Nb0.1O3	La0.6Sr0.4Fe0.7Ni0.3O3	SmAlO3
BaFe0.9Pr0.1O3	La0.6Sr0.4Fe0.85Mo0.15O3	SmBO3
BaFe0.9Y0.1O3	La0.6Sr0.4Fe0.8Cu0.2O3	SmCo0.5Mn0.5O3
BaFeO3	La0.6Sr0.4FeO3	SmCo0.95Ni0.05O3
BaGeO3	La0.6Tb0.2Ca0.2CoO3	SmCoO3
BaHfO3	La0.75Ca0.25Mn0.5Fe0.5O3	SmCrO3
BaIn0.75Sn0.25O3	La0.75Sr0.25Co0.5Mn0.5O3	SmFeO3
BaIn0.75Zr0.25O3	La0.75Sr0.25Cu0.25Co0.25Mn0.5O3	SmInO3
BaIrO3	La0.75Sr0.25Cu0.5Mn0.5O3	SmMnO3
BaMnO3	La0.75Sr0.25Mn0.5Cr0.2Al0.3O3	SmNiO3
BaMoO3	La0.75Sr0.25Mn0.5Cr0.3Al0.2O3	SmRhO3
BaNb0.05Fe0.95O3	La0.75Sr0.25Mn0.5Cr0.4Al0.1O3	SmScO3
BaNbO3	La0.75Sr0.25Mn0.5Cr0.5O3	SmSiO3
BaNiO3	La0.75Sr0.25Mn0.5Fe0.5O3	SmTiO3
BaPbO3	La0.7Ba0.3CoO3	SmVO3
BaPrO3	La0.7Ba0.3Mn0.1Co0.9O3	SmYO3
BaPuO3	La0.7Ba0.3Mn0.2Co0.8O3	SnTaO3
BaRhO3	La0.7Ba0.3Mn0.3Co0.1O3	Sr0.05Ca0.7Y0.25Co0.25Mn0.75O3
BaRuO3	La0.7Ba0.3Mn0.3Co0.7O3	Sr0.15Ca0.6Y0.25Co0.25Mn0.75O3
BaSc0.125Co0.875O3	La0.7Ba0.3Mn0.4Co0.6O3	Sr0.25Ca0.5Y0.25Co0.25Mn0.75O3
BaSc0.25Co0.75O3	La0.7Ba0.3Mn0.5Co0.5O3	Sr0.35Ca0.4Y0.25Co0.25Mn0.75O3
BaSc0.375Co0.625O3	La0.7Ba0.3Mn0.6Co0.4O3	Sr0.45Ca0.3Y0.25Co0.25Mn0.75O3
BaSiO3	La0.7Ba0.3Mn0.7Co0.3O3	Sr0.4La0.6Ti0.8Fe0.2O3

BaSn0.1Ti0.9O3	La0.7Ba0.3MnO3	Sr0.55Ca0.2Y0.25Co0.25Mn0.75O3
BaSnO3	La0.7Bi0.1Sr0.2FeO3	Sr0.65Ca0.1Y0.25Co0.25Mn0.75O3
BaTbO3	La0.7Ca0.05Pb0.25MnO3	Sr0.6La0.4Ti0.8Fe0.2O3
BaTeO3	La0.7Ca0.15Pb0.15MnO3	Sr0.75Bi0.25CoO3
BaTi0.05Fe0.95O3	La0.7Ca0.1Pb0.2MnO3	Sr0.75Ca0.25Fe0.85Ge0.15O3
BaTi0.5Fe0.33Mo0.17O3	La0.7Ca0.225Pb0.075MnO3	Sr0.75Ca0.25Fe0.95Ge0.05O3
BaTi0.5Fe0.33W0.17O3	La0.7Ca0.25Pb0.05MnO3	Sr0.75Ca0.25Fe0.9Ge0.1O3
BaTi0.93Ce0.07O3	La0.7Ca0.275Pb0.025MnO3	Sr0.75Ca0.25FeO3
BaTiO3	La0.7Ca0.2Ba0.1MnO3	Sr0.75Y0.25Co0.25Mn0.75O3
BaY0.667Mo0.333O3	La0.7Ca0.2Pb0.1MnO3	Sr0.75Y0.25Co0.5Mn0.5O3
BaY0.667W0.333O3	La0.7Ca0.3CoO3	Sr0.75Y0.25Co0.75Mn0.25O3
BaYb0.5Ta0.5O3	La0.7Ca0.3Cr0.2Ti0.8O3	Sr0.7Ho0.3CoO3
BaZr0.05Fe0.95O3	La0.7Ca0.3Cr0.5Ti0.5O3	Sr0.7Y0.3CoO3
BaZr0.125Yb0.125Co0.75O3	La0.7Ca0.3Fe0.8Ni0.2O3	Sr0.85Bi0.15CoO3
BaZr0.1Ce0.7Y0.2O3	La0.7Ca0.3Mn0.92Al0.08O3	Sr0.85Ce0.15CoO3
BaZr0.1Fe0.65Ni0.25O3	La0.7Ca0.3Mn0.94Al0.06O3	Sr0.8Bi0.2CoO3
BaZr0.1Fe0.75Ni0.15O3	La0.7Ca0.3Mn0.96Al0.04O3	Sr0.95Bi0.05CoO3
BaZr0.1Fe0.7Ni0.2O3	La0.7Ca0.3Mn0.98Al0.02O3	Sr0.95Ce0.05CoO3
BaZr0.1Fe0.8Ni0.1O3	La0.7Ca0.3Mn0.9Al0.1O3	Sr0.95K0.05Fe0.8Nb0.1Ta0.1O3
BaZr0.21Fe0.64Ni0.15O3	La0.7Ca0.3MnO3	Sr0.95Li0.05Fe0.8Nb0.1Ta0.1O3
BaZr0.25Yb0.125Co0.625O3	La0.7Na0.3MnO3	Sr0.95Na0.05Fe0.8Nb0.1Ta0.1O3
BaZr0.26Fe0.64Ni0.1O3	La0.7Pb0.3MnO3	Sr0.9Bi0.1CoO3
BaZr0.2Y0.1Fe0.7O3	La0.7Sr0.3Co0.25Fe0.25Mn0.25Ni0.25O3	Sr0.9Ce0.1Co0.9Nb0.1O3
BaZr0.31Fe0.64Ni0.05O3	La0.7Sr0.3Co0.2Fe0.6Zr0.2O3	Sr0.9Ce0.1CoO3
BaZr0.36Fe0.64O3	La0.7Sr0.3Co0.3Fe0.7O3	Sr0.9K0.1Fe0.1Co0.9O3
BaZr0.375Yb0.125Co0.5O3	La0.7Sr0.3Co0.3Mn0.7O3	Sr0.9K0.1Fe0.3Co0.7O3
BaZr0.5Yb0.125Co0.375O3	La0.7Sr0.3CoO3	Sr0.9K0.1Fe0.5Co0.5O3
BaZr0.625Yb0.125Co0.25O3	La0.7Sr0.3Fe0.7Ni0.3O3	Sr0.9K0.1Fe0.7Co0.3O3
BaZr0.75Yb0.125Co0.125O3	La0.7Sr0.3Ti0.1Fe0.6Ni0.3O3	Sr0.9K0.1Fe0.9Co0.1O3
BaZrO3	La0.83Ba0.17Fe0.5Cr0.5O3	Sr0.9K0.1FeO3

Bi0.15Sr0.85Co0.8Fe0.2O3	La0.83Ca0.17Fe0.5Cr0.5O3	SrCeO3
Bi0.4Sr0.6Co0.3Fe0.7O3	La0.85Ag0.15Mn0.5Co0.5O3	SrCo0.1Fe0.9O3
Bi0.5Ba0.5FeO3	La0.85Ag0.15Mn0.7Co0.3O3	SrCo0.2Fe0.6Nb0.2O3
Bi0.5Sr0.5Fe0.85Nb0.15O3	La0.85Ag0.15Mn0.9Co0.1O3	SrCo0.2Fe0.6Zr0.2O3
Bi0.5Sr0.5Fe0.85Ti0.15O3	La0.85Nd0.15Fe0.5Cr0.5O3	SrCo0.2Fe0.75Nb0.05O3
Bi0.5Sr0.5Fe0.85Zr0.15O3	La0.85Sr0.15Ni0.5Mn0.5O3	SrCo0.2Fe0.7Nb0.1O3
Bi0.5Sr0.5Fe0.8Co0.2O3	La0.8Ba0.1Ca0.1Mn0.95Co0.05O3	SrCo0.2Fe0.8O3
Bi0.5Sr0.5Fe0.8Cu0.2O3	La0.8Ba0.1Ca0.1Mn0.9Co0.1O3	SrCo0.3Fe0.7O3
Bi0.5Sr0.5Fe0.8Ti0.2O3	La0.8Ba0.1Ca0.1MnO3	SrCo0.4Fe0.4Zr0.1Y0.1O3
Bi0.5Sr0.5Fe0.90Zr0.10O3	La0.8Ca0.2CoO3	SrCo0.4Fe0.6O3
Bi0.5Sr0.5Fe0.95Nb0.05O3	La0.8Ca0.2Cr0.2Ti0.8O3	SrCo0.5Fe0.25Cu0.2Nb0.05O3
Bi0.5Sr0.5Fe0.95Nd0.05O3	La0.8Ca0.2Cr0.5Ti0.5O3	SrCo0.5Fe0.2Ti0.1Ta0.1Nb0.1O3
Bi0.5Sr0.5Fe0.95P0.05O3	La0.8Ca0.2Fe0.8Ni0.2O3	SrCo0.5Fe0.5O3
Bi0.5Sr0.5Fe0.95Sm0.05O3	La0.8Na0.2MnO3	SrCo0.67W0.33O3
Bi0.5Sr0.5Fe0.95Ta0.05O3	La0.8Nd0.2Fe0.5Cr0.5O3	SrCo0.6Fe0.25Sn0.15O3
Bi0.5Sr0.5Fe0.95Ti0.05O3	La0.8Sr0.2Co0.17Mn0.83O3	SrCo0.6Fe0.35Sn0.05O3
Bi0.5Sr0.5Fe0.95Zr0.05O3	La0.8Sr0.2Co0.1Fe0.9O3	SrCo0.6Fe0.375Sn0.025O3
Bi0.5Sr0.5Fe0.9Nb0.1O3	La0.8Sr0.2Co0.25Fe0.25Mn0.25Ni0.25O3	SrCo0.6Fe0.3Sn0.1O3
Bi0.5Sr0.5Fe0.9Sb0.1O3	La0.8Sr0.2Co0.2Fe0.4Nb0.4O3	SrCo0.6Fe0.4O3
Bi0.5Sr0.5Fe0.9Sn0.1O3	La0.8Sr0.2Co0.2Fe0.5Nb0.3O3	SrCo0.6Nb0.1Ni0.3O3
Bi0.5Sr0.5Fe0.9Ta0.1O3	La0.8Sr0.2Co0.2Fe0.6Nb0.2O3	SrCo0.6Ta0.4O3
Bi0.5Sr0.5Fe0.9Ti0.1O3	La0.8Sr0.2Co0.2Fe0.72Nb0.08O3	SrCo0.72Fe0.0.18Ta0.1O3
Bi0.5Sr0.5FeO3	La0.8Sr0.2Co0.2Fe0.74Nb0.06O3	SrCo0.75Nb0.05Cu0.2O3
Bi0.7Pr0.1Ba0.2FeO3	La0.8Sr0.2Co0.2Fe0.76Nb0.04O3	SrCo0.7Fe0.2Sc0.1O3
Bi0.7Sr0.3Fe0.925Ni0.075O3	La0.8Sr0.2Co0.2Fe0.78Nb0.02O3	SrCo0.7Fe0.2Ta0.1O3
Bi0.7Sr0.3Fe0.95Ni0.05O3	La0.8Sr0.2Co0.2Fe0.7Nb0.1O3	SrCo0.7Fe0.3O3
Bi0.7Sr0.3Fe0.975Ni0.025O3	La0.8Sr0.2Co0.2Fe0.8O3	SrCo0.7Nb0.1Fe0.2O3
Bi0.7Sr0.3Fe0.9Ni0.1O3	La0.8Sr0.2Co0.2Mn0.8O3	SrCo0.7Nb0.1Ni0.2O3
Bi0.7Sr0.3FeO3	La0.8Sr0.2Co0.3Fe0.7O3	SrCo0.85Fe0.15O3
Bi0.8Nd0.2FeO3	La0.8Sr0.2Co0.3Mn0.7O3	SrCo0.85Fe0.1P0.05O3

Bi0.8Pb0.2Fe0.8Ti0.2O3	La0.8Sr0.2Co0.4Fe0.6O3	SrCo0.85Ir0.15O3
Bi0.8Sr0.2FeO3	La0.8Sr0.2Co0.5Fe0.5O3	SrCo0.85Nb0.05Cu0.1O3
Bi0.9Nd0.1FeO3	La0.8Sr0.2Co0.6Fe0.4O3	SrCo0.85Nb0.15O3
BiAlO3	La0.8Sr0.2Co0.7Fe0.3O3	SrCo0.85Sb0.15O3
BiCo0.37Fe0.63O3	La0.8Sr0.2Co0.8Fe0.2O3	SrCo0.85Ta0.15O3
BiCo0.4Fe0.6O3	La0.8Sr0.2Co0.9Fe0.1O3	SrCo0.85Ti0.15O3
BiFe0.98Co0.02O3	La0.8Sr0.2CoO3	SrCo0.8Fe0.15P0.05O3
BiFeO3	La0.8Sr0.2Fe0.7Ni0.3O3	SrCo0.8Fe0.15Zr0.05O3
BiInO3	La0.8Sr0.2FeO3	SrCo0.8Fe0.1Ga0.1O3
BiMnO3	La0.8Sr0.2Mn0.85Sc0.15O3	SrCo0.8Fe0.1Nb0.1O3
BiScO3	La0.8Sr0.2Mn0.90Sc0.10O3	SrCo0.8Fe0.2O3
BiSmO3	La0.8Sr0.2Mn0.95Sc0.05O3	SrCo0.8Nb0.05Cu0.15O3
Ca0.06Mg0.68Fe0.26SiO3	La0.8Sr0.2MnO3	SrCo0.8Nb0.1Cu0.1O3
Ca0.1Mg0.34Fe0.56SiO3	La0.8Sr2Co0.2Fe0.6Zr0.2O3	SrCo0.8Nb0.1Ni0.1O3
Ca0.228Mn0.772SiO3	La0.95Sr0.05Ni0.5Mn0.5O3	SrCo0.8Nb0.1Ta0.1O3
Ca0.36Mn0.64SiO3	La0.9Ba0.1MnO3	SrCo0.8Nb0.2O3
Ca0.51Mn0.49SiO3	La0.9Ca0.1CoO3	SrCo0.8Sb0.2O3
Ca0.5Ce0.5MnO3	La0.9Ca0.1Cr0.2Ti0.8O3	SrCo0.8Ta0.1Cu0.1O3
Ca0.5Sr0.5MnO3	La0.9Ca0.1Cr0.5Ti0.5O3	SrCo0.8Ta0.2O3
Ca0.75Y0.25Co0.25Mn0.75O3	La0.9Ca0.1Fe0.8Ni0.2O3	SrCo0.8Ti0.2O3
Ca0.81Mn0.19SiO3	La0.9Na0.1MnO3	SrCo0.925Mo0.075O3
Ca0.85Zn0.15Fe0.5V0.5O3	La0.9Nd0.1Fe0.5Cr0.5O3	SrCo0.95Fe0.05O3
Ca0.95Zn0.05Fe0.5V0.5O3	La0.9Sr0.1Co0.1Mn0.9O3	SrCo0.95Mo0.05O3
Ca0.9Ag0.1Ti0.8Fe0.1Mg0.1O3	La0.9Sr0.1Co0.25Fe0.25Mn0.25Ni0.25O3	SrCo0.95Nb0.05O3
Ca0.9Bi0.1Ti0.9Fe0.1O3	La0.9Sr0.1Co0.2Fe0.6Zr0.2O3	SrCo0.95Sb0.05O3
Ca0.9Eu0.1Ti0.8Fe0.1Mg0.1O3	La0.9Sr0.1Co0.2Fe0.8O3	SrCo0.95Ta0.05O3
Ca0.9Pr0.1Ti0.8Fe0.1Mg0.1O3	La0.9Sr0.1Co0.2Mn0.8O3	SrCo0.95Ti0.05O3
Ca0.9Zn0.1Fe0.5V0.5O3	La0.9Sr0.1Co0.3Fe0.7O3	SrCo0.97V0.03O3

CaCO3

CaCr0.2Ti0.8O3

CaCr0.5Ti0.5O3

CaCrO3

CaFe0.5V0.5O3

CaGeO3

CaHfO3

CaIrO3

CaMn0.5Ce0.5O3

CaMn0.9Nb0.1O3

CaMnO3

CaMoO3

CaNbO3

CaPbO3

CaPtO3

CaRuO3

CaSiO3

CaSnO3

CaTi0.73Fe0.18Mg0.09O3

CaTi0.7Fe0.2Mg0.1O3

CaTi0.85Mo0.05Mg0.1O3

CaTi0.85Pd0.05Fe0.1O3

CaTi0.85V0.05Mg0.1O3

CaTi0.8Al0.1Fe0.1O3

CaTi0.8Co0.1Mg0.1O3

CaTi0.8Cr0.1Mg0.1O3

CaTi0.8Fe0.2O3

CaTi0.8Ga0.1Fe0.1O3

CaTi0.8Ga0.2O3

CaTi0.8Ge0.1Fe0.1O3

CaTi0.8Mg0.2O3

CaTi0.8Mn0.1Fe0.1O3

La0.9Sr0.1Co0.3Mn0.7O3

La0.9Sr0.1Fe0.7Ni0.3O3

La0.9Sr0.1Ni0.5Mn0.5O3

LaAlO3

LaBO3

LaCo0.25Fe0.25Mn0.25Ni0.25O3

LaCo0.25Mn0.75O3

LaCo0.42Fe0.58O3

LaCo0.4Ni0.3Cu0.3O3

LaCo0.4Ni0.4Cu0.2O3

LaCo0.4Ni0.5Cu0.1O3

LaCo0.4Ni0.6O3

LaCo0.5Fe0.5O3

LaCo0.5Mn0.5O3

LaCo0.71V0.29O3

LaCo0.7Cu0.3O3

LaCo0.8Cu0.2O3

LaCo0.95Ni0.05O3

LaCo0.9Cu0.1O3

LaCoO3

LaCr0.1 Ni0.9O3

LaCr0.2Ni0.8O3

LaCr0.2Ti0.8O3

LaCr0.3Mn0.7O3

LaCr0.3Ni0.7O3

LaCr0.4Ni0.6O3

LaCr0.5Ti0.5O3

LaCr0.7Ni0.3O3

LaCrO3

LaCu0.25Mn0.75O3

LaCuO3

LaDyO3

SrCo0.9Al0.1O3

SrCo0.9Fe0.1O3

SrCo0.9Ir0.1O3

SrCo0.9Mo0.1O3

SrCo0.9Nb0.05Cu0.05O3

SrCo0.9Nb0.1O3

SrCo0.9Sb0.1O3

SrCo0.9Ta0.1O3

SrCo0.9Ti0.1O3

SrCo0.9Y0.1O3

SrCO3

SrCoO3

SrCrO3

SrFe0.25Ti0.25Co0.25Mn0.25O3

SrFe0.45Co0.45Mo0.1O3

SrFe0.475Co0.475Mo0.05O3

SrFe0.475Co0.475Sb0.05O3

SrFe0.5Co0.5O3

SrFe0.5Ti0.2Co0.2Mn0.1O3

SrFe0.6Cu0.3W0.1O3

SrFe0.75Mo0.25O3

SrFe0.75Si0.25O3

SrFe0.7Cu0.2W0.1O3

SrFe0.7Cu0.3O3

SrFe0.7Si0.3O3

SrFe0.7W0.3O3

SrFe0.855Ta0.095P0.05O3

SrFe0.85Ge0.15O3

SrFe0.85Si0.15O3

SrFe0.85Ti0.15O3

SrFe0.8Al0.2O3

SrFe0.8Cu0.1W0.1O3

CaTi0.8Mn0.1Mg0.1O3
CaTi0.8Nb0.1Mg0.1O3
CaTi0.8Ni0.1Mg0.1O3
CaTi0.8V0.1Mg0.1O3
CaTi0.9Al0.1O3
CaTi0.9Bi0.1O3
CaTi0.9Co0.1O3
CaTi0.9Cu0.1O3
CaTi0.9Fe0.1O3
CaTi0.9Ga0.1O3
CaTi0.9Mg0.1O3
CaTi0.9Mn0.1O3
CaTi0.9Ni0.1O3
CaTi0.9Sb0.1O3
CaTi0.9Y0.1O3
CaTiO3
CaVO3
CaZrO3
CdCO3
CdGeO3
CdPbO3
CdSeO3
CdSnO3
CdTeO3
Ce0.1Sr0.9Co0.3Fe0.7O3
CeAlO3
CeBO3
CeCo0.5Mn0.5O3
CeCrO3
CeDyO3
CeErO3
CeFeO3

LaErO3
LaFe0.25Mn0.75O3
LaFe0.5Cr0.5O3
LaFe0.7Ni0.3O3
LaFe0.85Ti0.15O3
LaFe0.8Cu0.2O3
LaFe0.8Ni0.2O3
LaFe0.8Ti0.2O3
LaFe0.95Ti0.05O3
LaFe0.9Ti0.1O3
LaFeO3
LaGa0.2Co0.6Mg0.2O3
LaGa0.2Co0.8O3
LaGa0.3Co0.6Mg0.1O3
LaGa0.4Co0.35Mg0.25O3
LaGa0.4Co0.4Mg0.2O3
LaGa0.4Co0.6O3
LaGa0.5Co0.5O3
LaGa0.5Fe0.5O3
LaGa0.5Mn0.5O3
LaGa0.5Ni0.5O3
LaGa0.6Co0.4O3
LaGa0.8Co0.2O3
LaGaO3
LaHoO3
LaInO3
LaLuO3
LaMn0.5Fe0.5O3
LaMnO3
LaNi0.25Mn0.75O3
LaNi0.5Fe0.5O3
LaNi0.5Mn0.5O3

SrFe0.8Cu0.2O3
SrFe0.8Mo0.2O3
SrFe0.8Nb0.1Ta0.1O3
SrFe0.8Nb0.2O3
SrFe0.8Sc0.2O3
SrFe0.8Si0.2O3
SrFe0.8Ta0.1W0.1O3
SrFe0.8W0.2O3
SrFe0.90Si0.10O3
SrFe0.95Ge0.05O3
SrFe0.95Mo0.05O3
SrFe0.95Nb0.05O3
SrFe0.95Sc0.05O3
SrFe0.95Si0.05O3
SrFe0.95Ti0.05O3
SrFe0.9Al0.1O3
SrFe0.9Cu0.1O3
SrFe0.9Ge0.1O3
SrFe0.9Hf0.1O3
SrFe0.9Mo0.1O3
SrFe0.9Nb0.1O3
SrFe0.9Sc0.1O3
SrFe0.9Ti0.1O3
SrFe0.9W0.1O3
SrFeO3
SrGeO3
SrHfO3
SrIrO3
SrMnO3
SrMo0.2Fe0.8O3
SrMo0.9Co0.1O3
SrMoO3

CeGaO3

CeLuO3

CeMnO3

CeTiO3

CeTmO3

CeVO3

CeYbO3

Co0.224Mg0.776SiO3

CoGeO3

CoMnO3

CoSiO3

CoTeO3

CoTiO3

CrBO3

CsNbO3

CsVO3

CuCO3

CuNbO3

CuPO3

CuSiO3

CuTeO3

CuVO3

Dy0.1Sr0.9CoO3

Dy0.2Sr0.8CoO3

Dy0.3Sr0.7CoO3

Dy0.3Y0.7MnO3

Dy0.4Sr0.6CoO3

Dy0.5Y0.5MnO3

Dy0.7Y0.3MnO3

DyAlO3

DyBO3

DyCo0.5Mn0.5O3

LaNi0.6Fe0.4O3

LaNiO3

LaRhO3

LaRuO3

LaScO3

LaTiO3

LaTmO3

LaVO3

LaYbO3

LaYO3

Li0.12Na0.88NbO3

Li0.2Na0.8NbO3

Li0.96Na0.04NbO3

LiAsO3

LiBiO3

LiNbO3

LiPO3

LiReO3

LiSbO3

LiVO3

LuAlO3

LuBO3

LuCrO3

LuFeO3

LuMnO3

Mg0.25Nb0.49Ti0.26PbO3

Mg0.25Zn0.25Mn0.25Ni0.25TiO3

Mg0.2Zn0.8TiO3

Mg0.318Fe0.666Ca0.016SiO3

Mg0.39Fe0.52Ca0.09SiO3

Mg0.3Zn0.7TiO3

Mg0.46Mn0.54TiO3

SrNb0.05Ta0.15Fe0.8O3

SrNb0.15Ta0.05Fe0.8O3

SrNb0.1Co0.4Fe0.5O3

SrNb0.1Co0.6Fe0.3O3

SrNb0.1Co0.7Fe0.2O3

SrNb0.1Co0.8Fe0.1O3

SrNb0.1Co0.9O3

SrNb0.1Fe0.9O3

SrNb0.1Ta0.1Fe0.8O3

SrNb0.1W0.1Fe0.8O3

SrNb0.2Fe0.8O3

SrNbO3

SrPbO3

SrPrO3

SrPuO3

SrRuO3

SrSc0.025Nb0.075Fe0.9O3

SrSc0.025Ta0.075Fe0.9O3

SrSc0.02Co0.98O3

SrSc0.05Co0.95O3

SrSc0.05Ta0.05Fe0.9O3

SrSc0.075Ta0.025Fe0.9O3

SrSc0.175Nb0.025Co0.8O3

SrSc0.175Nb0.025Fe0.8O3

SrSc0.175Ta0.025Co0.8O3

SrSc0.1Co0.9O3

SrSc0.1Fe0.9O3

SrSc0.2Co0.8O3

SrSiO3

SrSnO3

SrTa0.05Nb0.15Fe0.8O3

SrTa0.15Nb0.05Fe0.8O3

DyCo0.95Ni0.05O3	Mg0.4Zn0.6TiO3	SrTa0.1Fe0.9O3
DyCoO3	Mg0.54Fe0.46SiO3	SrTa0.1Mo0.1Fe0.8O3
DyCrO3	Mg0.5Zn0.5TiO3	SrTa0.1Nb0.1Fe0.8O3
DyFeO3	Mg0.61Fe0.39SiO3	SrTa0.2Fe0.8O3
DyInO3	Mg0.69Fe0.23Ca0.08SiO3	SrTbO3
DyMnO3	Mg0.6Zn0.4TiO3	SrTi0.3Fe0.6Ni0.1O3
DyNiO3	Mg0.78Fe0.18Ca0.03Na0.02Si0.99Al0.005Fe0.005O3	SrTiO3
DyRhO3	Mg0.7Zn0.3TiO3	SrVO3
DyScO3	Mg0.8Zn0.2TiO3	SrW0.2Fe0.8O3
DyTiO3	Mg0.916Ca0.084SiO3	SrZrO3
DyVO3	Mg0.925Mn0.075SiO3	TbAlO3
ErAlO3	Mg0.944Ca0.056SiO3	TbCo0.5Mn0.5O3
ErBO3	Mg0.956Al0.044Al0.044Si0.956O3	TbCoO3
ErCrO3	Mg0.95Co0.05TiO3	TbCrO3
ErFeO3	Mg0.95Zn0.05TiO3	TbFeO3
ErMnO3	Mg0.962Cr0.038Si0.962Cr0.038O3	TbMnO3
ErNiO3	Mg0.972Fe0.028SiO3	TbRhO3
ErRhO3	Mg0.977Cr0.023Si0.977Cr0.023O3	TbTiO3
ErVO3	Mg0.985Cr0.015Si0.985Cr0.015O3	TbVO3
Eu0.5Sr0.5MnO3	Mg0.9Zn0.1TiO3	Ti0.2Mg0.27Nb0.53PbO3
Eu0.6Sr0.4MnO3	MgCO3	TiBO3
Eu0.7Sr0.3MnO3	MgGe0.21Si0.79O3	TlCrO3
Eu0.8Sr0.2MnO3	MgGe0.34Si0.66O3	TlFeO3
Eu0.9Sr0.1MnO3	MgGe0.3Si0.7O3	TlNiO3
EuAlO3	MgGe0.63Si0.37O3	TlSbO3
EuCo0.5Mn0.5O3	MgGe0.912Si0.088O3	TlVO3
EuCrO3	MgGeO3	TmAlO3
EuFeO3	MgSeO3	TmBO3
EuGaO3	MgSiO3	TmCrO3
EuInO3	MgSnO3	TmFeO3
EuMnO3	MgTiO3	TmNiO3

EuNbO3	MgVO3	TmTiO3
EuNiO3	Mn0.2Fe0.8TiO3	TmVO3
EuRhO3	Mn0.2Zn0.8TiO3	TmYO3
EuScO3	Mn0.32Ni0.68TiO3	VAlO3
EuTiO3	Mn0.3Zn0.7TiO3	VBO3
Fe0.155Mg0.845SiO3	Mn0.4Fe0.6TiO3	VCrO3
Fe0.2Mn0.8TiO3	Mn0.4Mg0.6TiO3	Y0.5Ca0.5Fe0.5Co0.5O3
Fe0.3Mg0.7SiO3	Mn0.5Fe0.5TiO3	Y0.7Ca0.3Fe0.5Co0.5O3
Fe0.4Mn0.6TiO3	Mn0.5Mg0.5TiO3	Y0.9Ca0.1Fe0.5Co0.5O3
Fe0.5Mn0.5TiO3	Mn0.5Ni0.5TiO3	Y0.9Ca0.1Fe0.6Co0.4O3
Fe0.6Co0.4TiO3	Mn0.5Zn0.5TiO3	Y0.9Ca0.1Fe0.7Co0.3O3
Fe0.6Mn0.4TiO3	Mn0.67Ni0.33TiO3	Y0.9Ca0.1Fe0.8Co0.2O3
Fe0.8Mn0.2TiO3	Mn0.6Fe0.4TiO3	YAlO3
FeBO3	Mn0.6Mg0.4TiO3	YbAlO3
FeTiO3	Mn0.7Co0.3GeO3	YbBO3
Ga0.5Al0.5FeO3	Mn0.7Fe0.3GeO3	YbCoO3
Ga0.75Al0.25FeO3	Mn0.7Mg0.3TiO3	YbCrO3
GaAlO3	Mn0.7Zn0.3TiO3	YbFeO3
GaBO3	Mn0.8Fe0.2TiO3	YbGaO3
GaFeO3	Mn0.8Mg0.2TiO3	YbMnO3
GaInO3	Mn0.9Mg0.1TiO3	YbNiO3
Gd0.5Ba0.5Mn0.5Fe0.5O3	Mn0.9Zn0.1TiO3	YBO3
Gd0.5Sr0.5Fe0.8Cu0.2O3	MnFe0.5Sb0.5O3	YbTiO3
Gd0.8Sr0.2Co0.2Fe0.8O3	MnGeO3	YbVO3
Gd0.8Sr0.2Co0.4Fe0.6O3	MnSeO3	YCo0.5Fe0.5O3
Gd0.8Sr0.2Co0.6Fe0.4O3	MnSiO3	YCo0.5Mn0.5O3
Gd0.8Sr0.2Co0.8Fe0.2O3	MnSnO3	YCoO3
Gd0.8Sr0.2CoO3	MnTiO3	YCrO3
Gd0.8Sr0.2FeO3	Na0.5Li0.5VO3	YFe0.5Co0.5O3
GdAlO3	Na0.84Li0.16VO3	YFeO3
GdBO3	NaAsO3	YGaO3
GdCo0.5Mn0.5O3	NaBiO3	YMnO3

GdCo0.95Ni0.05O3	NaIrO3	YNiO3
GdCrO3	NaNbO3	YScO3
GdFeO3	NaPO3	YTiO3
GdGaO3	NaSbO3	YVO3
GdMn0.7Ni0.3O3	NaTaO3	Zn0.333Mn0.333 Mg0.334TiO3
GdNiO3	NaVO3	Zn0.54Mg0.46TiO3
GdRhO3	NaWO3	ZnGeO3
GdScO3	Nd0.125Sr0.875Co0.875Ta0.125O3	ZnSiO3
GdTiO3	Nd0.1Ba0.9Co0.7Fe0.3O3	ZnSnO3
GdVO3	Nd0.2Sr0.8CoO3	ZnTeO3
HgSeO3	Nd0.2Sr0.8Nb0.1Co0.9O3	ZnTiO3
HgTeO3	Nd0.3Sr0.7CoO3	

Table S2. Elemental attributes used to construct the composition-based descriptors in this study[1].

Elemental Attributes		
Atomic Number (Z)	Melting Temperature	Heat Capacity Mass
Atomic Radii (Å)	Mendeleev Number	Heat Capacity Molar
Atomic Volume	Miracle Radius	Heat Fusion
Atomic Weight	Number[b]	Heat Vaporization
Boiling Temperature	Period	Second Ionization Energy
Bulk Modulus	Polarizability	Shear Modulus
Column[a]	Row[c]	Specific Heat Capacity
Covalent Radii	Elastic Modulus	Thermal Conductivity
Covalent Radius (pm)	Electrical Conductivity	Thermal Expansion Coefficient
Density	Electron Affinity	Third Ionization Energy
ICSD Volume	Electronegativity	n_ws^third ($(n_{ws})^{1/3}$)[d]
Ionic Radii	First Ionization Energy	phi[e]
Ionization Energy	Group	

Note: All elemental attributes were obtained from MAST-ML[1].

[a] Numerically identical to Group.

[b] Numerically identical to Atomic Number.

[c] Numerically identical to Period.

[d] The cube root of the electron density at the Wigner–Seitz cell boundary.

[e] The adjusted work function used in Miedema's model

Table S3. Hyperparameters for the final three modes for formability prediction.

Algorithm	Hyperparameters Tuned
Random Forest	'class_weight': 'balanced', 'max_depth': None, 'min_samples_leaf': 1, 'min_samples_split': 2, 'n_estimators': 300
XGBoost	'colsample_bytree': 0.9, 'learning_rate': 0.2, 'max_depth': None, 'n_estimators': 300, 'scale_pos_weight': 2, 'subsample': 0.9
LightGBM	'class_weight': None, 'colsample_bytree': 1.0, 'learning_rate': 0.1, 'max_depth': 10, 'n_estimators': 100, 'num_leaves': 31, 'subsample': 0.8

Table S4. Hyperparameters for the final three modes for cubic prediction.

Algorithm	Hyperparameters Tuned
Random Forest	'class_weight': 'balanced', 'max_depth': 10, 'min_samples_leaf': 1, 'min_samples_split': 5, 'n_estimators': 300
XGBoost	'colsample_bytree': 0.8, 'learning_rate': 0.1, 'max_depth': 7, 'n_estimators': 100, 'scale_pos_weight': 2, 'subsample': 1.0
LightGBM	'class_weight': None, 'colsample_bytree': 0.8, 'learning_rate': 0.1, 'max_depth': 10, 'n_estimators': 100, 'num_leaves': 31, 'subsample': 0.8

Table S5. Top 20 predicted formable and cubic perovskite oxides ranked by prediction probability.

Perovskite compounds	*t*	Formability	Cubic
Ba0.1Sr0.9Co0.3Fe0.6Ta0.1O2.60	0.992	1	1
Ba0.1Sr0.9Co0.3Fe0.6Ta0.1O2.90	1.010	1	1
Ba0.1Sr0.9Co0.3Fe0.6Ta0.1O2.75	1.004	1	1
Sr1Co0.1Fe0.7Mo0.1Nb0.1O2.70	0.984	1	1
Sr1Co0.1Fe0.7Mo0.1Nb0.1O2.75	0.985	1	1
Sr1Co0.1Fe0.7Mo0.1Nb0.1O2.75	0.988	1	1
Sr1Co0.1Fe0.7Mo0.1Nb0.1O2.80	0.989	1	1
Sr1Co0.2Fe0.6Nb0.1Ta0.1O2.70	0.984	1	1
Sr1Co0.3Fe0.5Nb0.1Ta0.1O2.70	0.986	1	1
Sr1Co0.4Fe0.4Nb0.1Ta0.1O2.70	0.988	1	1
Sr1Co0.2Fe0.6Nb0.1Ta0.1O3.00	1.002	1	1
Sr1Co0.3Fe0.5Nb0.1Ta0.1O2.95	1.001	1	1
Sr1Co0.4Fe0.4Nb0.1Ta0.1O2.90	1.000	1	1
Sr1Co0.2Fe0.6Nb0.1Ta0.1O2.80	0.992	1	1
Sr1Co0.3Fe0.5Nb0.1Ta0.1O2.85	0.998	1	1
Sr1Co0.4Fe0.4Nb0.1Ta0.1O2.90	1.004	1	1
Sr1Co0.5Fe0.3Mo0.1Nb0.1O2.70	0.992	1	1
Sr1Co0.5Fe0.3Mo0.1Nb0.1O2.75	0.993	1	1
Sr1Co0.5Fe0.3Mo0.1Nb0.1O2.85	1.001	1	1
Sr1Co0.5Fe0.3Mo0.1Nb0.1O2.90	1.002	1	1

1. Jacobs, R.; Mayeshiba, T.; Afflerbach, B.; Miles, L.; Williams, M.; Turner, M.; Finkel, R.; Morgan, D., The Materials Simulation Toolkit for Machine learning (MAST-ML): An automated open source toolkit to accelerate data-driven materials research. *Comput. Mater. Sci.* **2020,** 176, 109544.
